# Assessment of the quality of notifications and investigations of congenital syphilis in Bahia, 2010–2021

**DOI:** 10.1590/1980-220X-REEUSP-2025-0582en

**Published:** 2026-09-07

**Authors:** Jeane Magnavita da Fonseca Cerqueira, Maria Aparecida Araújo Figueiredo, Nila Mara Smith Galvão, Monique Magnavita Borba da Fonseca Cerqueira, Magno Merces Weyll Pimentel

**Affiliations:** 1 Universidade do Estado da Bahia, Departamento de Ciência da Vida, Salvador, BA, Brazil.; 2 Universidade do Estado da Bahia, Departamento de Ciências Exatas e da Terra, Salvador, BA, Brazil.

**Keywords:** Syphilis, Congenital, Epidemiologic Surveillance Services, Disease Notification, Epidemiology, Health Information Systems.

## Abstract

**Objective:**

To evaluate the quality of the information in the congenital syphilis notification and investigation forms as a tool for epidemiological surveillance to monitor children with the disease.

**Methods:**

An ecological time-trend study was conducted using secondary data from the SINAN system in Bahia between 2010 and 2021. The study assessed the annual percentages of incompleteness, the proportional percentage variation, and the percentage of inconsistency. The temporal trend in incompleteness was analyzed using Spearman’s correlation coefficient.

**Results:**

Among the ten maternal variables evaluated, a reduction in data incompleteness was observed for seven of them. In the fields pertaining to the child, there was a reduction in the degree of data incompleteness for nine of the 11 variables evaluated (p ≤ 0.05). In the child follow-up, the study found that 86.7% of the investigation forms did not contain information on the nontreponemal test in peripheral blood at birth. For the treponemal test after 18 months, the percentage of forms lacking this information was 99.3%.

**Conclusion:**

Despite the reduction in the number of incomplete fields on the congenital syphilis investigation form, the high percentage of forms lacking information on laboratory results compromises the assessment of clinical progression and follow-up of children with syphilis.

## INTRODUCTION

Congenital syphilis (CS), which is transmitted vertically, is considered the most serious outcome of the infection due to the potential complications for the child. Diagnosis is challenging, as many children are born asymptomatic or present with mild, nonspecific clinical signs^(1)^. This characteristic undermines control strategies, delaying the start of treatment and making it difficult to provide adequate follow-up^(2,3)^.

The evaluation of a child exposed to or affected by CS should begin immediately after birth, with clinical, epidemiological, and laboratory investigations, and should continue until the child is two years old or, at a minimum, until 18 months of age^(2,3)^. Longitudinal follow-up of all children is essential to confirm a definitive cure and break the cycle of transmission. However, the lack of highly accurate diagnostic tests for newborns represents one of the main obstacles to effective monitoring^(4)^. In addition, a complex series of laboratory tests, particularly nontreponemal tests (NTT) on peripheral blood, is needed, and their proper execution is essential for a definitive diagnosis. Failure to perform these tests compromises epidemiological understanding and may have implications for the child’s health in later stages^(5)^.

In CS, continuous care requires integration across the various points in the child health care network^(5)^. A structured care pathway enables qualified follow-up, with epidemiological surveillance playing a strategic role in the identification, reporting, investigation, and monitoring of CS cases^(6)^. Since care for children with CS begins with understanding the case, CS has been included on the list of reportable diseases nationwide in Brazil since 1986. The Notifiable Diseases Information System (SINAN, as per its acronym in Portuguese) is the primary tool for collecting, processing, and distributing information on reportable diseases, based on records from health services^(7)^. Thus, monitoring reported cases until the investigation is concluded generates epidemiological data that inform timely policies and actions^(8)^.

In this regard, the effectiveness of CS surveillance depends on the quality of the information, as inconsistent data undermine the reliability of indicators and the implementation of evidence-based measures^(9,10)^. Properly completing notifications is an essential step in generating valid information and is a direct result of the work carried out by healthcare professionals in their day-to-day practice^(6)^.

In the field of public health, nurses play a key role in integrating surveillance with primary health care (PHC) due to their generalist training and their role in organizing teams, coordinating care, providing clinical management, and conducting epidemiological follow-up of syphilis cases—all of which require technical and scientific rigor, mastery of protocols, and ethical responsibility in recording information. In this context, the improper completion of notification and investigation forms (NIFs) can introduce bias into information systems, hinder understanding of the health-disease process, and impede the adoption of effective strategies. Since CS is an indicator of the quality of prenatal and maternal-child care, flaws in these records can result in underreporting, compromise data reliability, and directly impact the practice of primary care nurses^(10,11)^.

Within the context of CS, the ongoing analysis of SINAN data is essential for obtaining an accurate picture of the epidemiological situation and for the effective planning of public health interventions. Among the main criteria for assessing the reliability of the data produced, the following stand out: incompleteness, which refers to the failure to provide necessary information; and inconsistency, which relates to the coherence among interrelated fields^(10)^. Thus, the lack of quality monitoring for this data underscores the need for more in-depth investigations^(12)^.

With this in mind, this study aims to evaluate the quality of the information contained in the CS notification and investigation forms as a tool for epidemiological surveillance in the follow-up of children affected by the disease.

## METHOD

### Study Type

This is an ecological, time-series study of reported and investigated cases of CS in the state of Bahia between 2010 and 2021.

The study followed the recommendations of the Reporting of Studies Conducted Using Observational Routinely-Collected Health Data (RECORD statement), an extension of the Strengthening the Reporting of Observational Studies in Epidemiology (STROBE) statement, aimed at standardization and transparency in the conduct and reporting of studies using secondary data routinely collected in health information systems^(13,14)^.

### Study Location

State of Bahia, Brazil, using secondary data provided by the Epidemiological Surveillance Division of the Bahia State Department of Health (SESAB, as per its acronym in Portuguese).

### Population and Selection Criteria

All confirmed cases of CS recorded in SINAN from January 2010 to July 2021 were included, in accordance with the current national definition. CS is a notifiable disease, including cases involving newborns, stillbirths, or abortions in women with untreated or inadequately treated syphilis. Treatment is considered adequate when administered with benzathine penicillin, according to the clinical stage, and initiated no later than 30 days before delivery^(2)^.

For surveillance purposes, CS is defined as: I) cases in children under 13 years of age who present with at least one of the following: clinical, cerebrospinal fluid, or radiological manifestations of the disease with a positive NTT; NTT titers higher than the mother’s by two dilutions; rising NTT titers by two dilutions during follow-up of the exposed child; a positive NTT after six months of age, even with adequate neonatal treatment; or a positive treponemal test (TT) after 18 months, without a prior diagnosis of CS, excluding acquired syphilis; II) a child up to two years of age with microbiological evidence of Treponema pallidum infection, detected in nasal secretions, a skin lesion, a biopsy or autopsy of the child, a miscarriage, or a stillbirth, through direct examination using dark-field microscopy or stained material^(2)^.

### Sample Definition or Sample Size Calculation

Since this was a census-based study using secondary data, no sample calculation was performed. All available records from the period were analyzed.

### Data Collection

The CS NIF has 66 fields, divided between pregnant women/mothers and children. The pregnant women/mothers section has 17 fields, divided into epidemiological history, laboratory data, and treatment. The children’s section includes 20 fields, organized into epidemiological history, laboratory and clinical data, treatment, and clinical course.

The maternal variables analyzed were: age, race/ethnicity, occupation, educational level, participation in prenatal care, diagnosis of syphilis, nontreponemal test (NTT), and treponemal test (TT) at delivery/curettage, treatment regimen, and treatment of sexual partners. With regard to the child, the following were considered: municipality of residence, NTT in peripheral blood, whether an NTT was performed, and the date of the NTT after 18 months, NTT in cerebrospinal fluid (CSF), ascending NTT titers, CSF abnormalities, radiological and clinical diagnosis, signs and symptoms, treatment regimen, clinical course, and date of death.

### Data Analysis and Processing

Inconsistent records were not excluded, as they were the subject of the study’s analysis. Duplicate records were identified based on the variables “mother’s name” and “child’s year of birth,” with only one record retained per case. Records related to twin pregnancies were retained in the database, as they corresponded to distinct events.

Operationally, a field was considered incomplete if it was blank or marked as “ignored”; fields categorized as “not performed” were considered filled in but analyzed separately, as they represented a failure in care.

The following scoring system was used to classify data incompleteness: ≤ 5% of fields incomplete (satisfactorily complete); 5.1–10% (slightly incomplete); 10.1–30% (moderately incomplete); 30.1–50% (very incomplete); and >50% (highly incomplete). Once classified, data quality was assessed based on the following parameters: i) annual incompleteness rate (proportion of unfilled fields per year); ii) proportional percentage variation (PPV), calculated as the proportional change in the absolute number of unfilled fields between the first and last years of the series; iii) percentage of inconsistency (cases in which the completion of a variable that depends on a related variable was omitted or entered incorrectly).

The temporal trend in incompleteness was analyzed using Spearman’s correlation coefficient (r_s_), a nonparametric method suitable for analyzing temporal trends in proportions of incompleteness, which does not require a normal distribution of the data. Considering a statistical significance level of p ≤ 0.05, the trend was classified as: i) increasing when r_s_ was positive; ii) decreasing when r_s_ was negative; iii) stationary when r_s_ was close to zero.

To analyze inconsistencies, the relationship between the following fields was examined: i) the child’s clinical diagnosis marked as asymptomatic; not applicable; or ignored versus whether the field regarding the presence of signs and symptoms was filled out; ii) the child’s clinical diagnosis marked as symptomatic versus the presence of signs and symptoms marked as not applicable or ignored; iii) a treponemal test performed after 18 months marked as reactive or nonreactive versus the date the test was performed before 18 months of age (excluding abortions, stillbirths, and deaths).

The analyses were performed using Stata® version 15.0.

### Ethical Considerations

The study was approved by the Research Ethics Committee of the State University of Bahia (Opinion No. 7,619,184, CAAE 38510920.6.0000.0057), with a waiver of informed consent due to the use of secondary data from institutional sources. The study was funded by the Bahia State Research Support Foundation (FAPESB), Call for Proposals 02/2020.

### Use of Artificial Intelligence

This manuscript made occasional use of the generative artificial intelligence tool ChatGPT (https://chat.openai.com), limited to the analysis of textual coherence and fluency. No scientific content or references were generated by the tool, and the analyses, interpretations, and conclusions were developed entirely by the authors, with the references verified in their full versions.

## RESULTS

In the state of Bahia, from January 2010 to July 2021, 11,602 CS NIFs were reported and investigated. An analysis of the trend in the incompleteness of these NIFs and the PPV between the first and last years of the series revealed different patterns and degrees of incompleteness.

During the years under study, a trend analysis of the degree of incompleteness for ten maternal variables related to children with CS revealed a decrease in seven of them, particularly in the following categories: age (PPV = –5.88; r_s_ = –0,76), race/ethnicity (PPV = –42.42; r_s_ = –0,68), prenatal care (PPV = –25.00; r_s_ = –0,80), diagnosis during prenatal care, delivery, or the postpartum period (PPV = –13.63; r_s_ = –0,60), TT at delivery (PPV = –36.11; r_s_ = –0,87), treatment of sexual partners (PPV = –12.50; r_s_ = –0,68), and treatment of the mother (PPV = –28.57; r_s_ = –0,51). With the exception of the latter, all other trends mentioned were statistically significant (p ≤ 0.05) (Table 1).

**Table 1:** Trends in data incompleteness and proportional percentage variation between the first and last years of the data series for variables related to mothers in congenital syphilis investigation forms – Bahia, Brazil, January 2010–July 2021.

Variables	2010	2011	2012	2013	2014	2015	2016	2017	2018	2019	2020	2021	PPV	r_s_	*p–value*
	Incompleteness rate														
Age	5.36	11.54	10.19	7.54	5.68	4.00	3.84	3.63	2.62	3.68	1.84	4.41	–5.88	–0.76	0.004
Race/color	20.82	28.74	24.60	18.86	10.31	12.69	12.85	15.75	17.64	17.96	9.58	10.47	–42.42	–0.68	0.015
Occupation	41.96	38.87	31.63	34.31	42.39	43.19	41.97	45.72	37.76	39.93	36.87	40.77	11.27	0.07	0.820
Education	41.96	41.50	40.42	45.50	43.55	42.33	36.73	30.77	33.87	39.01	41.34	42.15	15.03	–0.32	0.307
Implementation of PN	20.19	20.04	21.09	21.41	16.09	19.85	11.45	10.89	8.50	8.77	10.22	13.22	–25.00	–0.80	0.000
Diagnosis of syphilis in PN, childbirth, and postpartum	13.88	13.56	16.17	11.44	8.00	8.01	8.24	7.26	5.88	6.43	9.58	10.47	–13.63	–0.60	0.038
High-quality NTT in childbirth	5.68	7.49	9.14	6.93	4.91	4.68	6.08	5.30	4.79	3.76	2.63	4.96	0.00	–0.70	0.011
TT in childbirth	22.71	19.84	24.25	22.14	15.51	19.51	19.13	15.53	12.33	11.53	15.24	12.67	–36.11	–0.87	0.000
Mother’s treatment	26.50	17.41	24.78	20.19	15.51	14.99	15.64	14.95	19.81	15.96	15.16	16.53	–28.57	–0.51	0.080
Treatment of sexual partners	37.85	32.19	41.65	45.26	28.42	29.22	27.44	13.06	12.97	27.32	27.45	28.93	–12.50	–0.68	0.010

Note: PN – Prenatal; NTT – Nontreponemal test; TT – Treponemal test; PPV – Proportional Percentage Variation; *r*_s_ – Spearman’s correlation coefficient.

As for the levels of incompleteness analyzed by year, the age variable remained only slightly incomplete (5.1% to 10%), except in 2011; race/color was moderately incomplete (10.1% to 30%), decreasing from 20.82% to 15.59%; and occupation and maternal education remained very incomplete (30.1% to 50%) throughout the entire series. Prenatal care was moderately incomplete (10.1% to 30%), becoming slightly incomplete in 2018 and 2019 (5.1% to 10%); however, the diagnosis of maternal syphilis improved starting in 2014, classified as slightly incomplete (5.1% to 10%). The NTT fields during delivery were slightly incomplete (5.1% to 10%) in most years, reaching a satisfactorily complete level (≤ 5%) in 2014 and 2015 and from 2018 to 2021, but the TT fields remained moderately incomplete (10.1% to 30%). Treatment for the mother ranged from moderately incomplete (10.1% to 30%) to slightly incomplete (5.1% to 10%), while treatment of sexual partners ranged from very incomplete (30.1% to 50%) to moderately incomplete (10.1% to 30%) starting in 2014 (Table 1). The variables related to the child’s epidemiological history were satisfactorily complete (<5%) for the epidemiological week, date the report was completed, age, and sex (data not shown).

An analysis of the trend in data incompleteness for 11 variables related to the epidemiological history of children with CS showed that, between 2010 and 2021, there was a consistent reduction in the degree of incompleteness for nine of these variables, all of which were statistically significant (p ≤ 0.05), namely: abnormal CSF test results (VPP = –26,03; r_s_ = –0,60), NTT in CSF (VPP = –41,35; r_s_ = –0,91), NTT in peripheral blood (VPP = –48,80; r_s_ = –0,71), clinical diagnosis (VPP = –53,03; r_s_ = –0,94), radiological diagnosis (VPP = –27,50; r_s_ = –0,93), treatment regimen (VPP = –21,21; r_s_ = –0,92), TT after 18 months (VPP = –30,46; r_s_ = –0,84), date of TT after 18 months (VPP = –28,77; r_s_ = –0,88), and date of death (VPP = –37,04; r_s_ = –0,60) (Table 2).

**Table 2:** Trends in incompleteness and proportional percentage variation between the first and last years of the data series for child-related variables in congenital syphilis investigation forms – Bahia, Brazil, January 2010–July 2021.

Variables	2010	2011	2012	2013	2014	2015	2016	2017	2018	2019	2020	2021	PPV	*r*_s_	*p–value*
	Incompleteness rate													
Place of birth	5.36	19.23	24.25	17.40	15.13	12.86	9.71	12.26	10.42	4.18	3.99	5.23	0.00	–0.70	0.011
Changes in the CSF test	46.06	49.39	53.95	47.81	43.93	36.97	31.22	33.02	31.05	27.07	21.39	29.75	–26.03	–0.60	0.000
NTT in CSF	41.96	45.95	49.74	44.04	42.00	35.35	27.58	28.74	25.69	25.06	17.16	21.49	–41.35	–0.91	0.000
NTT in the blood	12.93	28.95	30.40	22.99	18.40	17.89	14.53	15.67	13.74	8.10	8.06	11.85	–48.80	–0.71	0.009
Clinical diagnosis	41.64	49.19	40.42	30.29	28.71	21.21	21.72	22.06	19.23	14.12	13.97	17.08	–53.03	–0.94	0.000
Radiological diagnosis	50.47	52.92	58.52	49.88	49.52	40.20	32.40	34.62	34.12	27.74	25.70	31.96	–27.50	–0.93	0.000
Treatment plan	91.70	29.96	32.51	25.19	21.39	19.08	18.23	18.51	17.89	11.19	11.89	14.60	–21.21	–0.92	0.000
TT after 18 months	40.38	45.14	48.33	42.94	36.03	32.96	24.09	25.54	28.95	29.32	18.91	24.52	–30.46	–0.84	0.000
TT date after 18 months	43.85	46.36	49.74	46.11	43.74	39.86	28.07	27.50	30.86	31.24	22.19	27.27	–28.77	–0.88	0.000
Progress	15.14	14.98	17.05	17.60	15.30	9.20	10.27	15.46	14.38	9.02	11.09	9.09	–31.25	–0.50	0.095
Date of death	17.03	17.00	19.68	19.46	16.74	10.82	11.38	16.55	15.08	9.36	11.97	9.37	–37.04	–0.80	0.039

Note: NTT – Nontreponemal test; TT – Treponemal test; PPV – Proportional Percentage Variation; r_s_ – Spearman’s correlation coefficient.

Analysis of the degree of incompleteness by year showed that the “municipality of residence” field ranged from slightly incomplete (5.1% to 10%) in 2010, 2019, 2020, and 2021 to moderately incomplete (10.1% to 30%) in the other years. NTT in cerebrospinal fluid was very incomplete (30.1% to 50%) until 2015, subsequently decreasing to moderately incomplete (10.1% to 30%). In contrast, NTT in peripheral blood was considered very incomplete (30.1% to 50%) in most years, except in 2019 and 2020, when it became slightly incomplete (5.1% to 10%). The “CSF abnormalities” field remained very incomplete (30.1% to 50%) in most years, reaching highly incomplete (>50%) in 2012; however, in the last three years of the series, it changed to moderately incomplete (10.1% to 30%). Radiological diagnosis was highly incomplete during the first three years (>50%), becoming very incomplete (30.1% to 50%) over the next six years, and moderately incomplete (5.1% to 10%) in 2019 and 2020. The treatment regimen remained moderately incomplete (10.1% to 30%) for most of the period. TT at 18 months ranged from very incomplete (30.1% to 50%) between 2010 and 2015 to moderately incomplete (10.1% to 30%) in the following years. The date of TT after 18 months ranged from very incomplete (30.1% to 50%) to moderately incomplete (10.1% to 30%). The “case progression” field alternated between moderately incomplete (10.1% to 30%) and slightly incomplete (5.1% to 10%), while the “date of death” field was moderately incomplete (10.1% to 30%) for most of the period and slightly incomplete (5.1% to 10%) in three of the 12 years studied. The “signs and symptoms” field for children with CS ranged from moderately incomplete (10.1% to 30%), very incomplete (30.1% to 50%), and highly incomplete (>50%). The “asymptomatic” variable ranged from very incomplete (30.1% to 50%) to moderately incomplete (10.1% to 30%) (Table 2).

Table 3 shows the number of signs and symptoms recorded in the congenital syphilis investigation forms, according to clinical diagnosis. Of the 11,602 records analyzed, 61.45% (7,130/11,602) were diagnosed as asymptomatic, 10% (1,161/11,602) as symptomatic, 5.06% (588/11,602) as “not applicable,” and 13.05% (1,515/11,602) as unknown or blank. Among those classified as symptomatic (1,161), 21.27% (247/1,161) had no recorded signs or symptoms. Among those classified as asymptomatic, six had recorded signs and symptoms.

**Table 3:** Number of signs and symptoms recorded in congenital syphilis investigation forms, by clinical diagnosis – Bahia, Brazil, January 2010–July 2021.

Clinical diagnosis	0	1	2	3	4	5	6	7	8	Total
Asymptomatic	7,124	6	0	0	0	0	0	0	0	7,130
Symptomatic	247	707	110	53	24	10	3	3	4	1,161
Not applicable	588	0	0	0	0	0	0	0	0	588
Ignored	1,515	0	0	0	0	0	0	0	0	1,515
Blank	1,208	0	0	0	0	0	0	0	0	1,208
Total	10,682	713	110	53	24	10	3	3	4	11,602

Source: SINAN.

The first part of Table 4 compares the NTT level in peripheral blood at birth with the values entered in the “ascending titration” field in subsequent tests, which should be performed at 1, 3, 6, 12, and 18 months. Of the 8,360 CS NIF records for living children, 4,765 did not undergo the test for subsequent titration, and 2,484 had an unknown status, totaling 86.7% of CS NIF records lacking this information. The second part of Table 4 shows the status of the TT field for tests performed after 18 months, considering the child’s age on the date of the test. Among the 8,178 CS NIF cases in living children, 8,121 (99.3%) had no information regarding whether the test was performed, two underwent the test before 18 months of age, and 56 (0.7%) underwent the test at the recommended age (over 18 months).

**Table 4:** Completion of the “ascending titers” field related to the nontreponemal test and the status of the treponemal test after 18 months, according to the child’s age on the date the test was performed, in the congenital syphilis investigation forms – Bahia, Brazil, January 2010–July 2021.

Nontreponemal test in peripheral blood	Ascending titers				
	Yes	No	Not performed	Ignored	Blank	Total
Reactive	94	1,017	4,765	2,484	0	8.360
Non-reactive	4	68	455	166	0	693
Not performed	7	11	619	85	0	722
Ignored	6	18	154	443	0	621
Blank	0	0	0	0	1,206	1.206
Total	111	1,114	5,993	3,178	1,206	11.602
**Treponemal test after 18 months**	**Age of the children alive at the time of the test**				
	**<18 months**	**18 to 24 months**		**>24 months**	**Blank**	**Total**
Reactive	0		1	4	162	167
Non-reactive	2		26	24	139	191
Not performed	0		0	0	2,418	2,418
Not applicable	0		0	0	3,097	3,097
Ignored	0		0	0	1,576	1,576
Blank	0		0	0	729	729
Total	2		27	28	8,121	8,178

Source: SINAN.

## DISCUSSION

In the state of Bahia, the trend of incompleteness in the CS NIF data from January 2010 to July 2021 decreased for most of the variables analyzed. However, inconsistencies persisted in variables essential for monitoring children with the infection, especially those related to laboratory tests that are indispensable for clinical and laboratory follow-up and for evaluating therapeutic response. These findings point to weaknesses in the quality of records and limit the monitoring of the clinical and laboratory progression of cases.

The improvement in the completeness of some variables over the course of the time series may be associated with changes in the organization of syphilis surveillance in Brazil, the strengthening of public policies aimed at eliminating vertical transmission, and improvements in surveillance and care—including the implementation of the Clinical Protocol and Therapeutic Guidelines (PCDT, as per its acronym in Portuguese) for Comprehensive Care for People with Sexually Transmitted Infections and the National Pact for the Elimination of Vertical Transmission of HIV, Syphilis, Hepatitis B, and Chagas Disease as a public health problem^(2)^. These initiatives, combined with the expansion of primary health care (PHC) — particularly through the Family Health Strategy — its coordination with health surveillance, the progressive computerization of services, the increased use of SINAN, and the consolidation of routines for data entry, review, and monitoring, may have contributed to the more timely identification of cases, the longitudinal follow-up of children with CS, increased awareness among health professionals, and the adoption of more systematic recording practices, thereby contributing to a reduction in missing or blank fields and to the gradual improvement in the quality of information^(6,7)^. Although it is not possible to establish a causal relationship between these initiatives and the observed results, the findings suggest that changes in the organization of services and in the work processes of public health surveillance and health care may have played an important role in improving the completeness of records over the period analyzed.

In this regard, the production of high-quality information is an essential prerequisite for supporting evidence-based health analyses^(9)^. In the case of CS, a preventable disease whose elimination is among Brazil’s international commitments, coordinated responses are needed that integrate early diagnosis, timely treatment, and rigorous follow-up of newborns who are exposed or affected^(3)^. However, the effectiveness of these responses depends on the quality of the records kept by healthcare professionals, particularly nurses, who play a central role in conducting surveillance in primary health care.

Despite progress in clinical and laboratory variables, such as maternal diagnosis and tests at delivery, the performance of the NTT in the mother still requires attention. Although the rate of incomplete testing is low, this variable is essential for comparison with the child’s nontreponemal serology, guiding diagnostic, therapeutic, and surveillance decisions. Its absence compromises clinical management and the effectiveness of the health system’s response to congenital syphilis^(3)^.

The “maternal treatment” variable showed a reduction in data incompleteness between the extreme years, but the percentages remained high, especially when compared to the NTT. These findings highlight persistent gaps in information essential for the diagnosis and clinical management of newborns and children^(15)^. High levels of missing data also persisted in fields such as occupation and maternal education, which are essential for characterizing the sociodemographic profile of pregnant women. The absence or inadequacy of this information hinders the monitoring of maternal health and limits the implementation of strategies targeting the most vulnerable populations^(2,16)^.

Although the Ministry of Health has, since 2017, ceased to consider treatment of the sexual partner as a criterion for defining cases of congenital syphilis for reporting purposes, this change has not eliminated the need to follow up on all children who have been exposed to or have congenital syphilis^(17)^. In this study, information on sexual partners remained frequently incomplete throughout the time series, representing a significant gap, since the concurrent treatment of the mother and her sexual partners is essential for interrupting the transmission of *Treponema pallidum* and preventing reinfection^(18)^. Low adherence to treatment regimens, combined with a lack of awareness about the disease, failure by health services to provide treatment, and institutional challenges, constitutes a major obstacle to controlling vertical transmission, as it perpetuates the risk of reinfection and may compromise the timely and appropriate treatment of pregnant women. Thus, the absence or inadequacy of this information reduces the analytical capacity of information systems to monitor vertical transmission^(3)^.

Variables related to child care showed high percentages of blank or missing fields, especially in records regarding CSF, postnatal laboratory tests, X-rays of long bones, and treatment regimens, highlighting significant limitations in the completeness of this information. These gaps limit the assessment of the definitive diagnosis, the treatment prescribed, follow-up, and the individualization of care, in addition to compromising the surveillance system’s ability to monitor the clinical management of cases and potential late manifestations, which are often silent or nonspecific^(19)^.

Given this scenario, the incompleteness of CSF and laboratory test records takes on particular significance for the surveillance of congenital neurosyphilis, a severe and difficult-to-detect form of the disease, especially in asymptomatic newborns. Because it can manifest at different stages of infection and with varying signs, it requires a high degree of clinical suspicion, even in the absence of obvious neurological manifestations^(20)^. Therefore, a thorough evaluation, timely diagnosis, and accurate documentation of the treatment plan are essential to ensure the child’s proper management and follow-up and to identify early on situations that require intervention, thereby helping to prevent neurological, auditory, and psychomotor developmental disorders^(17)^. The literature emphasizes that comprehensive information is essential for the proper clinical and epidemiological characterization of health conditions and for informing the evaluation of health initiatives and services^(21)^.

From the perspective of inconsistency, discrepancies were observed between interdependent variables, such as the relationship between clinical diagnosis and the presence of signs and symptoms in children, which may reflect flaws in data recording or difficulties in applying the case definition criteria.

With regard to child follow-up, data from the NIFs play a strategic role as a surveillance tool for controlling the condition. However, this study revealed a high degree of incompleteness in some key information: the NTT test in peripheral blood, which should be performed at 1, 3, 6, 12, and 18 months of age, was missing from 86.7% of the NIFs, and for the TT test after 18 months, 99.3% of the NIF forms lacked this information. According to the Ministry of Health’s PCDT^(3)^, NTT tests, such as the Venereal Disease Research Laboratory (VDRL) test, are essential for monitoring infants after birth, as they allow for confirmation of the diagnosis and monitoring of the response to treatment by quantifying antibody titers^(2,4)^. Thus, the lack of data on subsequent increases in NTT levels makes it difficult to determine whether there were errors in data recording or whether the test was not performed. Similarly, the lack of information on TT after 18 months may reflect follow-up failures, limitations in data recording, or both, making it difficult to confirm the diagnosis of CS, as recommended by the ministerial protocol^(3)^.

In addition, two limitations of this tool for monitoring children’s follow-up were identified: the absence of specific fields for recording the NTT at each of the time points in the series recommended by the Ministry of Health (1, 3, 6, 12, and 18 months of age) and the use of the “not applicable” option in the variable regarding the performance of the TT after 18 months, since this test is recommended for confirming or ruling out infection in the child and is required by the current protocol for follow-up. These weaknesses may compromise the effectiveness of NIFs as a surveillance tool for monitoring children with CS.

Regarding the study’s limitations, the possibility of reporting bias must be considered, as incomplete fields may reflect either recording errors or the procedure not having been performed, with no way to distinguish between the two based on the information available in the system. It should also be noted that the data were collected through July 2021, the last year of the analyzed time series. However, no policies or interventions were identified that could have significantly altered reporting patterns in the subsequent months; therefore, the results remain representative.

## CONCLUSION

The study evaluated the quality of CS NIFs as a tool for epidemiological surveillance to monitor children affected by congenital syphilis. The results indicated a reduction in the incompleteness of the CS NIF fields, but persistent inconsistencies among interdependent variables and a high degree of incompleteness in the fields related to laboratory results, compromising the assessment of clinical progression, follow-up, and the adequacy of the investigation of children with CS.

These limitations appear to stem not only from gaps in record-keeping but also from structural shortcomings in the notification and investigation form, whose cross-sectional approach is insufficient to account for follow-up extending from birth to 18 months of age. Although the NIFs include variables related to the monitoring of congenital syphilis, such as ascending NTT titers and TT results after 18 months, they do not allow for the systematic recording of all follow-up stages recommended by clinical care protocols. Furthermore, the indiscriminate use of the “not applicable” category in the clinical diagnosis and treponemal test fields after 18 months makes it difficult to distinguish between situations of clinical non-indication, failure to perform the test, and interruption of follow-up, compromising data interpretation and the identification of follow-up losses. Taken together, these limitations reduce the analytical potential of epidemiological surveillance and restrict the use of the information for case monitoring, action planning, and evaluation of strategies to control congenital syphilis.

In light of this, there is a clear need for structural strategies to improve the reporting and investigation of CS, including ongoing training for teams, systematic supervision of records, and strengthening the integration between health surveillance, primary care, and other components of the healthcare network. The results also indicate the need to improve the SINAN form by incorporating mechanisms that allow for the recording of the different stages of serological monitoring and by revising the rules for filling out the “not applicable” category.

Finally, it should be emphasized that the quality of reporting and investigation is essential for information systems to support epidemiological surveillance, longitudinal care, and health decision-making. In this regard, interoperability between systems and the use of integrated digital solutions, in line with the principles of the Digital SUS, are fundamental strategies for strengthening surveillance, improving the quality of care, and expanding the health system’s capacity to respond to congenital syphilis.

## Data Availability

The data used were collected from SESAB’s Epidemiological Surveillance unit, as part of the Mother Project, from the SINAN nominal database, and pertained to cases of CS in the state between 2010 and July 2021. The analyses followed the statistical methodologies described in this manuscript.
