# Supplementary material for: Assessment of the quality of notifications and investigations of congenital syphilis in Bahia, 2010–2021
Source: Rev Esc Enferm USP. 2026 Sep 7;60:e20250582. doi: 10.1590/1980-220X-REEUSP-2025-0582en (PMC13577234; doi:10.1590/1980-220X-REEUSP-2025-0582en)
Supplement: Translation

# Translation: Avaliação da qualidade das notificações e investigações da sífilis congênita na Bahia, 2010–2021

**DOI:** 10.1590/1980-220X-REEUSP-2025-0582pt

**Authors:** Jeane Magnavita da Fonseca Cerqueira, Maria Aparecida Araújo Figueiredo, Nila Mara Smith Galvão, Monique Magnavita Borba da Fonseca Cerqueira, Magno Merces Weyll Pimentel

**Affiliations:** 1 Universidade do Estado da Bahia, Departamento de Ciência da Vida, Salvador, BA, Brasil.; 2 Universidade do Estado da Bahia, Departamento de Ciências Exatas e da Terra, Salvador, BA, Brasil.

**Keywords:** Sífilis Congênita, Serviços de Vigilância Epidemiológica, Notificação de Doenças, Epidemiologia, Sistemas de Informação em Saúde

## Abstract

**Objetivo:**

Avaliar a qualidade das informações das fichas de notificação e investigação da sífilis congênita como instrumento da vigilância epidemiológica para seguimento das crianças com a doença.

**Métodos:**

Estudo ecológico, de tendência temporal, com dados secundários do SINAN da Bahia, entre 2010 e 2021. Avaliaram-se os percentuais de incompletude anual, a variação percentual proporcional e o percentual de inconsistência. A tendência temporal da incompletude foi analisada pelo coeficiente de correlação de Spearman.

**Resultados:**

Dentre dez variáveis maternas avaliadas, observou-se redução da incompletude em sete delas. Nos campos referentes à criança, houve redução dos graus de incompletude em nove das 11 variáveis avaliadas (p ≤ 0,05). No seguimento da criança, o estudo evidenciou que 86,7% das fichas de investigação não continham informações do teste não treponêmico em sangue periférico ao nascer. Para o teste treponêmico após 18 meses, o percentual de fichas sem essa informação foi de 99,3%.

**Conclusão:**

Apesar da redução da incompletude dos campos da ficha de investigação de sífilis congênita, os altos percentuais de fichas sem informações sobre resultados laboratoriais comprometem a avaliação da evolução e do seguimento da criança com sífilis.

## INTRODUÇÃO

A Sífilis Congênita (SC), transmitida verticalmente, é considerada o desfecho mais grave da infecção em razão das possíveis complicações para a criança. O diagnóstico é desafiador, pois muitas crianças nascem assintomáticas ou apresentam sinais clínicos leves e inespecíficos^(1)^. Essa característica compromete as estratégias de controle, atrasando o início do tratamento e dificultando o acompanhamento adequado^(2,3)^.

A avaliação da criança exposta ou acometida por SC deve-se iniciar imediatamente após o nascimento, com investigação clínica, epidemiológica e laboratorial, mantida até os dois anos de idade ou, no mínimo, até os 18 meses^(2,3)^. O seguimento longitudinal de todas as crianças é fundamental para confirmar a cura definitiva e interromper o ciclo de transmissão. No entanto, a ausência de exames com alta acurácia para o diagnóstico em recém-nascidos representa um dos principais entraves ao monitoramento efetivo^(4)^. Além disso, soma-se a necessidade de realizar uma série complexa de testes laboratoriais, especialmente os Testes Não Treponêmicos (TNT) em sangue periférico, cuja execução adequada é essencial para o diagnóstico definitivo. A descontinuidade desses exames compromete a compreensão epidemiológica e pode trazer implicações à saúde da criança em fases posteriores^(5)^.

Na SC, o cuidado contínuo exige integração entre os diversos pontos da rede de atenção à saúde da criança^(5)^. Uma linha de cuidado estruturada viabiliza o seguimento qualificado, com a vigilância epidemiológica exercendo papel estratégico na identificação, notificação, investigação e monitoramento dos casos de SC^(6)^. Como o cuidado à criança com SC começa a partir do conhecimento do caso, no Brasil, a SC integra a lista de doenças de notificação compulsória em todo o território nacional, desde 1986. O Sistema de Informação de Agravos de Notificação (SINAN) é o principal instrumento para coleta, processamento e distribuição de informações sobre doenças notificáveis, com base nos registros dos serviços de saúde^(7)^. Dessa forma, o acompanhamento dos casos notificados até o encerramento da investigação gera dados epidemiológicos que orientam políticas e ações oportunas^(8)^.

Nesse sentido, a eficácia da vigilância da SC depende da qualidade das informações, pois dados inconsistentes comprometem a confiabilidade dos indicadores e a implementação de medidas baseadas em evidências^(9,10)^. O adequado preenchimento das notificações constitui etapa essencial para a produção de informações válidas, sendo resultado direto do trabalho dos profissionais de saúde no cotidiano do serviço^(6)^.

No âmbito da saúde pública, o enfermeiro destaca-se na integração entre vigilância e Atenção Primária à Saúde (APS), pela formação generalista e atuação na organização das equipes, condução do cuidado, pelo manejo clínico e acompanhamento epidemiológico dos casos de sífilis, o que exige rigor técnico-científico, domínio dos protocolos e responsabilidade ética no registro das informações. Nesse cenário, o preenchimento inadequado das Fichas de Notificação e Investigação (FNI) pode introduzir vieses nos sistemas de informação, dificultar a compreensão do processo saúde-doença e a adoção de estratégias eficazes. Por se tratar de indicador da qualidade do pré-natal e do cuidado materno-infantil, falhas nesses registros podem resultar em subnotificação, comprometer a confiabilidade dos dados e repercutir diretamente na prática do enfermeiro da APS^(10,11)^.

No âmbito da SC, a análise contínua dos dados do SINAN é fundamental para uma visão fidedigna da situação epidemiológica e para o planejamento efetivo das ações em saúde pública. Dentre os principais critérios para avaliar a confiabilidade dos dados produzidos, destacam-se a incompletude, que se refere ao não preenchimento de informações necessárias, e a inconsistência, que está relacionada à coerência entre campos interrelacionados^(10)^. Assim, a deficiência no monitoramento da qualidade desses dados reforça a necessidade de investigações mais aprofundadas^(12)^.

Com essa perspectiva, este estudo tem como objetivo avaliar a qualidade das informações das fichas de notificação e investigação da SC como instrumento da vigilância epidemiológica no seguimento da criança acometida pela doença.

## MÉTODO

### Tipo do Estudo

Trata-se de um estudo ecológico, de tendência temporal, dos casos de SC notificados e investigados no estado da Bahia, entre 2010 e 2021.

O estudo seguiu as recomendações do *Reporting of studies Conducted using Observational Routinely-collected health Data* (RECORD *statement*), uma extensão da declaração *Strengthening the Reporting of Observational Studies in Epidemiology* (STROBE), voltada à padronização e transparência na condução e no relato de estudos com dados secundários rotineiramente coletados em sistemas de informação em saúde^(13,14)^.

### Local do Estudo

Estado da Bahia, Brasil, utilizando dados secundários disponibilizados pela Vigilância Epidemiológica da Secretaria da Saúde do Estado da Bahia (SESAB).

### População e Critério de Seleção

Foram incluídos todos os casos confirmados de SC registrados no SINAN no período de janeiro de 2010 a julho de 2021, conforme definição nacional vigente. A SC é de notificação compulsória, incluindo casos de recém-nascidos, natimortos ou abortos de mulheres com sífilis não tratada ou tratada de forma inadequada. O tratamento é considerado adequado quando realizado com penicilina benzatina, conforme o estágio clínico, iniciado até 30 dias antes do parto^(2)^.

Para fins de vigilância, considera-se SC: I) casos em crianças com menos de 13 anos que apresentem pelo menos uma das seguintes situações: manifestação clínica, liquórica ou radiológica da doença com TNT reagente; títulos do TNT superiores aos da mãe em duas diluições; títulos do TNT ascendentes em duas diluições no seguimento da criança exposta; TNT reagente após seis meses de idade, mesmo com tratamento neonatal adequado; ou Teste Treponêmico (TT) reagente após 18 meses, sem diagnóstico prévio de SC, excluindo sífilis adquirida; II) criança de até dois anos com evidência microbiológica de infecção por *Treponema pallidum*, detectada em secreção nasal, lesão cutânea, biópsia ou necropsia de criança, aborto ou natimorto, por meio de exames diretos com microscopia de campo escuro ou material corado^(2)^.

### Definição da Amostra ou Cálculo do Tamanho da Amostra

Por se tratar de estudo censitário com base em dados secundários, não foi realizado cálculo amostral. Foram analisados todos os registros disponíveis no período.

### Coleta de Dados

A FNI da SC possui 66 campos, distribuídos entre gestantes/mães e crianças. O segmento da gestante/mãe possui 17 campos, distribuídos em antecedentes epidemiológicos, dados laboratoriais e tratamento. A seção da criança inclui 20 campos, organizados em antecedentes epidemiológicos, dados laboratoriais e clínicos, tratamento e evolução.

As variáveis maternas analisadas foram: idade, raça/cor, ocupação, escolaridade, realização de pré-natal, diagnóstico de sífilis, TNT e TT no parto/curetagem, esquema de tratamento e tratamento de parceiros. Em relação às crianças, foram consideradas: município de residência, TNT em sangue periférico, realização e data do TNT após 18 meses, TNT no líquor cefalorraquidiano (LCR), titulação ascendente do TNT, alteração liquórica, diagnóstico radiológico e clínico, sinais e sintomas, esquema terapêutico, evolução e data do óbito.

### Análise e Tratamento dos Dados

Registros inconsistentes não foram excluídos por constituírem objeto de análise do estudo. A identificação de duplicidades foi realizada com base nas variáveis nome da mãe e ano de nascimento da criança, mantendo-se apenas um registro por caso. Registros referentes a gestações gemelares foram mantidos no banco de dados, por corresponderem a eventos distintos.

Operacionalmente, considerou-se incompleto quando o campo estava em branco ou com código “ignorado”; campos com categoria “não realizado” foram considerados preenchidos, porém analisados separadamente, por representarem falha de cuidado.

Para classificação da incompletude, adotou-se o seguinte escore: ≤ 5% dos campos incompletos (satisfatoriamente completo); de 5,1 a 10% (pouco incompleto); de 10,1 a 30% (moderadamente incompleto); de 30,1 a 50% (muito incompleto) e >50% (altamente incompleto). Com base nessa classificação, a qualidade dos dados foi avaliada a partir dos seguintes parâmetros: i) percentual de incompletude anual (proporção de campos não preenchidos por ano); ii) Variação Percentual Proporcional (VPP) calculada pela variação proporcional do número absoluto de campos não preenchidos no primeiro e no último ano da série; iii) percentual de inconsistência (casos em que o preenchimento de uma variável que depende de outra relacionada foi omitido ou preenchido de forma incorreta).

A tendência temporal da incompletude foi analisada por meio do coeficiente de correlação de Spearman (*r*_S_), método não paramétrico adequado à análise de tendência temporal de proporções de incompletude, sem exigência de distribuição normal dos dados. Considerando-se a significância estatística de *p* ≤ 0,05, a tendência foi classificada como: i) crescente quando o valor do r_s_ foi positivo; ii) decrescente quando o valor do *r*_s_ foi negativo; e iii) estacionária quando o valor do *r*_s_ esteve próximo de zero.

Para a análise da inconsistência, foi observada a relação entre os seguintes campos: i) diagnóstico clínico da criança assinalado como assintomático; não se aplica; ou ignorado *versus* preenchimento do campo de presença de sinais e sintomas; ii) diagnóstico clínico da criança assinalado como sintomático *versus* presença de sinais e sintomas, marcado como não se aplica ou ignorado; iii) realização do teste treponêmico após 18 meses assinalado como reagente ou não reagente *versus* a data de realização do teste antes dos 18 meses de idade (exceto abortos, natimortos e óbitos).

As análises foram realizadas no *software Stata*® versão 15.0.

### Aspectos Éticos

O estudo foi aprovado pelo Comitê de Ética em Pesquisa da Universidade do Estado da Bahia (Parecer nº 7.619.184, CAAE 38510920.6.0000.0057), com dispensa do Termo de Consentimento Livre e Esclarecido por utilizar dados secundários de domínio institucional e financiado pela Fundação de Amparo à Pesquisa do Estado da Bahia (FAPESB), Edital 02/2020.

### Uso de Inteligência Artificial

Este manuscrito contou com o uso pontual da ferramenta de inteligência artificial generativa ChatGPT (https://chat.openai.com), restrita à análise de coerência e fluidez textual. Não houve geração de conteúdo científico ou referências, e as análises, interpretações e conclusões foram integralmente elaboradas pelos autores, com verificação das referências em suas versões completas.

## RESULTADOS

No estado da Bahia, no período de janeiro de 2010 a julho de 2021, foram notificados e investigados 11.602 FNI de SC. A análise da tendência da incompletude dessas FNI e a VPP entre o primeiro e o último ano da série revelaram diferentes padrões e graus de incompletude.

Nos anos observados, a análise de tendência do grau de incompletude de dez variáveis maternas referentes às crianças com SC evidenciou redução em sete delas, destacando-se os seguintes campos: idade (VPP = –5,88; r_s_ = –0,76), raça/cor (VPP = –42,42; r_s_ = –0,68), realização do pré-natal (VPP = –25,00; r_s_ = –0,80), diagnóstico no pré-natal, parto ou puerpério (VPP = –13,63; r_s_ = –0,60), TT no parto (VPP = –36,11; r_s_ = –0,87), tratamento das parcerias sexuais (VPP = –12,50; r_s_ = –0,68) e tratamento da mãe (VPP = –28,57; r_s_ = –0,51). Exceto esta última, todas as demais tendências citadas apresentaram significância estatística (p ≤ 0,05) (Tabela 1).

**Tabela 1:** Tendência da incompletude e variação percentual proporcional entre o primeiro e o último ano da série das variáveis relacionadas às mães, nas fichas de investigação de sífilis congênita – Bahia, Brasil, janeiro de 2010 a julho de 2021.

Variáveis	2010	2011	2012	2013	2014	2015	2016	2017	2018	2019	2020	2021	VPP	*r*_s_	Valor de *p*
	Percentual de incompletude														
Idade	5,36	11,54	10,19	7,54	5,68	4,00	3,84	3,63	2,62	3,68	1,84	4,41	–5,88	–0,76	0,004
Raça/cor	20,82	28,74	24,60	18,86	10,31	12,69	12,85	15,75	17,64	17,96	9,58	10,47	–42,42	–0,68	0,015
Ocupação	41,96	38,87	31,63	34,31	42,39	43,19	41,97	45,72	37,76	39,93	36,87	40,77	11,27	0,07	0,820
Escolaridade	41,96	41,50	40,42	45,50	43,55	42,33	36,73	30,77	33,87	39,01	41,34	42,15	15,03	–0,32	0,307
Realização do PN	20,19	20,04	21,09	21,41	16,09	19,85	11,45	10,89	8,50	8,77	10,22	13,22	–25,00	–0,80	0,000
Diagnóstico de sífilis no PN/parto/puerpério	13,88	13,56	16,17	11,44	8,00	8,01	8,24	7,26	5,88	6,43	9,58	10,47	–13,63	–0,60	0,038
TNT qualitativo no parto	5,68	7,49	9,14	6,93	4,91	4,68	6,08	5,30	4,79	3,76	2,63	4,96	0,00	–0,70	0,011
TT no parto	22,71	19,84	24,25	22,14	15,51	19,51	19,13	15,53	12,33	11,53	15,24	12,67	–36,11	–0,87	0,000
Tratamento da mãe	26,50	17,41	24,78	20,19	15,51	14,99	15,64	14,95	19,81	15,96	15,16	16,53	–28,57	–0,51	0,080
Tratamento das parcerias sexuais	37,85	32,19	41,65	45,26	28,42	29,22	27,44	13,06	12,97	27,32	27,45	28,93	–12,50	–0,68	0,010

Nota: PN – Pré-natal; TNT – Teste Não Treponêmico; TT – Teste Treponêmico; VPP – Variação Percentual Proporcional; *r*_s_ – Coeficiente de correlação de Spearman.

Quanto aos graus de incompletude analisados por ano, a variável idade manteve-se pouco incompleta (de 5,1% a 10%), exceto em 2011; raça/cor foi moderadamente incompleta (de 10,1% a 30%), reduzindo de 20,82% para 15,59%; e ocupação e escolaridade materna permaneceram muito incompletas (de 30,1% a 50%) durante toda a série. A realização do pré-natal foi moderadamente incompleta (de 10,1% a 30%), tornando-se pouco incompleta em 2018 e 2019 (de 5,1% a 10%); porém, o diagnóstico de sífilis materna melhorou a partir de 2014, classificando-se como pouco incompleto (de 5,1% a 10%). Os campos referentes ao TNT no parto foram pouco incompletos (de 5,1% a 10%) na maioria dos anos, atingindo grau satisfatoriamente completo (≤ 5%) em 2014 e 2015 e de 2018 a 2021, mas os campos do TT mantiveram-se moderadamente incompletos (de 10,1% a 30%). O tratamento da mãe variou de moderadamente incompleto (de 10,1% a 30%) a pouco incompleto (de 5,1% a 10%), enquanto o tratamento da parceria evoluiu de muito incompleto (de 30,1% a 50%) a moderadamente incompleto (de 10,1% a 30%), a partir de 2014 (Tabela 1). As variáveis de antecedentes epidemiológicos da criança apresentaram grau satisfatoriamente completo (< 5%) nos campos semana epidemiológica, data de preenchimento da notificação, idade e sexo (dados não demonstrados).

A análise da tendência de incompletude de 11 variáveis relacionadas aos antecedentes epidemiológicos da criança com SC mostrou que, entre 2010 e 2021, houve uma redução consistente nos graus de incompletude em nove delas, todas com significância estatística (p ≤ 0,05), a saber: alteração do exame do LCR (VPP = –26,03; r_s_ = –0,60), TNT no LCR (VPP = –41,53; r_s_ = –0,91), TNT no sangue periférico (VPP = –48,80; r_s_ = –0,71), diagnóstico clínico (VPP = –53,03; r_s_ = –0,94), diagnóstico radiológico (VPP = –27,50; r_s_ = –0,93), esquema de tratamento (VPP = –21,21; r_s_ = –0,92), TT após 18 meses (VPP = –30,46; r_s_ = –0,84), data do TT após 18 meses (VPP = –28,77; r_s_ = –0,88), data do óbito (VPP = –37,04; r_s_ = –0,60) (Tabela 2).

**Tabela 2:** Tendência da incompletude e variação percentual proporcional entre o primeiro e o último ano da série do preenchimento das variáveis relacionadas à criança, nas fichas de investigação da sífilis congênita – Bahia, Brasil, janeiro de 2010 a julho de 2021.

Variáveis	2010	2011	2012	2013	2014	2015	2016	2017	2018	2019	2020	2021	VPP	*r*_s_	Valor de *p*
	Percentual de incompletude														
Local de nascimento	5,36	19,23	24,25	17,40	15,13	12,86	9,71	12,26	10,42	4,18	3,99	5,23	0,00	–0,70	0,011
Alterações no exame do LCR	46,06	49,39	53,95	47,81	43,93	36,97	31,22	33,02	31,05	27,07	21,39	29,75	–26,03	–0,60	0,000
TNT em LCR	41,96	45,95	49,74	44,04	42,00	35,35	27,58	28,74	25,69	25,06	17,16	21,49	–41,35	–0,91	0,000
TNT em sangue	12,93	28,95	30,40	22,99	18,40	17,89	14,53	15,67	13,74	8,10	8,06	11,85	–48,80	–0,71	0,009
Diagnóstico clínico	41,64	49,19	40,42	30,29	28,71	21,21	21,72	22,06	19,23	14,12	13,97	17,08	–53,03	–0,94	0,000
Diagnóstico radiológico	50,47	52,92	58,52	49,88	49,52	40,20	32,40	34,62	34,12	27,74	25,70	31,96	–27,50	–0,93	0,000
Esquema de tratamento	91,70	29,96	32,51	25,19	21,39	19,08	18,23	18,51	17,89	11,19	11,89	14,60	–21,21	–0,92	0,000
TT após 18 meses	40,38	45,14	48,33	42,94	36,03	32,96	24,09	25,54	28,95	29,32	18,91	24,52	–30,46	–0,84	0,000
Data do TT após 18 meses	43,85	46,36	49,74	46,11	43,74	39,86	28,07	27,50	30,86	31,24	22,19	27,27	–28,77	–0,88	0,000
Evolução	15,14	14,98	17,05	17,60	15,30	9,20	10,27	15,46	14,38	9,02	11,09	9,09	–31,25	–0,50	0,095
Data do óbito	17,03	17,00	19,68	19,46	16,74	10,82	11,38	16,55	15,08	9,36	11,97	9,37	–37,04	–0,80	0,039

Nota: TNT – Teste Não Treponêmico; TT – Teste Treponêmico; VPP – Variação Percentual Proporcional; *r*_s_ – Coeficiente de correlação de Spearman.

A análise do grau de incompletude por ano demonstrou que o campo município de residência variou entre pouco incompleto (de 5,1% a 10%) nos anos 2010, 2019, 2020 e 2021 e moderadamente incompleto (de 10,1% a 30%) nos demais anos. O TNT no LCR cefalorraquidiano apresentou grau muito incompleto (de 30,1% a 50%) até 2015, reduzindo posteriormente para moderadamente incompleto (de 10,1% a 30%). Já o TNT em sangue periférico foi considerado muito incompleto (de 30,1% a 50%) na maioria dos anos, exceto em 2019 e 2020, quando passou a pouco incompleto (de 5,1% a 10%). O campo alterações no LCR manteve-se muito incompleto (de 30,1% a 50%) na maioria dos anos, chegando a altamente incompleto (> 50%) em 2012; todavia, nos três últimos anos da série, passou para moderadamente incompleto (de 10,1% a 30%). O diagnóstico radiológico foi altamente incompleto nos três primeiros anos (> 50%), passando para muito incompleto (de 30,1% a 50%) nos seis anos seguintes e moderadamente incompleto (de 5,1% a 10%) em 2019 e 2020. O esquema de tratamento manteve-se moderadamente incompleto (de 10,1% a 30%) na maior parte do período. O TT após 18 meses variou de muito incompleto (de 30,1% a 50%) entre 2010 e 2015 para moderadamente incompleto (de 10,1% a 30%) nos anos seguintes. A data de realização do TT após 18 meses variou entre muito incompleto (de 30,1% a 50%) e moderadamente incompleto (de 10,1% a 30%). O campo evolução do caso alternou entre moderadamente incompleto (de 10,1% a 30%) e pouco incompleto (de 5,1% a 10%), enquanto o campo data do óbito foi moderadamente incompleto (de 10,1% a 30%) na maior parte do período e pouco incompleto (de 5,1% a 10%) em três dos 12 anos estudados. O campo sinais e sintomas das crianças com SC oscilou entre moderadamente incompleto (de 10,1% a 30%), muito incompleto (de 30,1% a 50%) e altamente incompleto (> 50%). O campo da variável assintomático oscilou entre muito incompleto (de 30,1% a 50%) e moderadamente incompleto (de 10,1% a 30%) (Tabela 2).

A Tabela 3 apresenta o número de sinais e sintomas registrados nas fichas de investigação de sífilis congênita, segundo o diagnóstico clínico. Dos 11.602 registros analisados, 61,45% (7.130/11.602) foram diagnosticados como assintomáticos, 10% (1.161/11.602) como sintomáticos, 5,06% (588/11.602) como “não se aplica” e 13,05% (1.515/11.602) como ignorado ou em branco. Entre os classificados como sintomáticos (1.161), 21,27% (247/1.161) não tinham sinais e sintomas registrados. Entre os classificados como assintomáticos, seis apresentavam sinais e sintomas registrados.

**Tabela 3:** Número de sinais e sintomas registrados nas fichas de investigação de sífilis congênita, por diagnóstico clínico – Bahia, Brasil, janeiro de 2010 a julho de 2021.

Diagnóstico clínico	0	1	2	3	4	5	6	7	8	Total
Assintomático	7.124	6	0	0	0	0	0	0	0	7.130
Sintomático	247	707	110	53	24	10	3	3	4	1.161
Não se aplica	588	0	0	0	0	0	0	0	0	588
Ignorado	1.515	0	0	0	0	0	0	0	0	1.515
Em branco	1.208	0	0	0	0	0	0	0	0	1.208
Total	10.682	713	110	53	24	10	3	3	4	11.602

Fonte: SINAN.

A primeira parte da Tabela 4 compara o TNT no sangue periférico ao nascer com o preenchimento do campo “titulação ascendente” nos testes subsequentes, que devem ser realizados nos meses 1, 3, 6, 12 e 18. Das 8.360 FNI de SC em crianças vivas, 4.765 não realizaram o teste para observação da titulação subsequente e 2.484 estavam com situação ignorada, totalizando 86,7% das FNI de SC sem essa informação. Na segunda parte da Tabela 4, observa-se a situação do campo do TT realizado após 18 meses, considerando a idade da criança na data do exame. Dentre as 8.178 FNI de SC em crianças vivas, 8.121 (99,3%) não tinham informação sobre a realização do teste, duas realizaram o teste antes dos 18 meses e 56 (0,7%) realizaram o teste na idade recomendada (acima de 18 meses).

**Tabela 4:** Preenchimento do campo titulação ascendente relacionado ao teste não treponêmico e situação do teste treponêmico após 18 meses, segundo a idade da criança na data de realização do teste, nas fichas de investigação de sífilis congênita – Bahia, Brasil, janeiro de 2010 a julho de 2021.

Teste não treponêmico em sangue periférico	Titulação ascendente					
	Sim	Não	Não realizado	Ignorado	Em branco	Total
Reagente	94	1.017	4.765	2.484	0	8.360
Não reagente	4	68	455	166	0	693
Não realizado	7	11	619	85	0	722
Ignorado	6	18	154	443	0	621
Em branco	0	0	0	0	1.206	1.206
Total	111	1.114	5.993	3.178	1.206	11.602
**Teste treponêmico após 18 meses**	**Idade das crianças vivas no teste**					
	**<18 meses**		**18 a 24 meses**	**>24 meses**	**Em branco**	**Total**
Reagente	0		1	4	162	167
Não reagente	2		26	24	139	191
Não realizado	0		0	0	2.418	2.418
Não se aplica	0		0	0	3.097	3.097
Ignorado	0		0	0	1.576	1.576
Em branco	0		0	0	729	729
Total	2		27	28	8.121	8.178

Fonte: SINAN.

## DISCUSSÃO

No estado da Bahia, a tendência da incompletude das FNI de SC, de janeiro de 2010 a julho de 2021, reduziu-se na maioria das variáveis analisadas. Entretanto, persistiram inconsistências em variáveis essenciais ao acompanhamento da criança com infecção, especialmente as relacionadas aos testes laboratoriais indispensáveis ao seguimento clínico-laboratorial e à avaliação da resposta terapêutica. Esses achados apontam fragilidades na qualidade dos registros e limitam o seguimento da evolução clínica e laboratorial dos casos.

A melhora da completude de parte das variáveis ao longo da série histórica pode estar associada a mudanças na organização da vigilância da SC no Brasil, ao fortalecimento das políticas públicas voltadas à eliminação da transmissão vertical e à qualificação da vigilância e da assistência, incluindo a implementação do Protocolo Clínico e Diretrizes Terapêuticas (PCDT) para Atenção Integral às Pessoas com Infecções Sexualmente Transmissíveis e do Pacto Nacional para a Eliminação da Transmissão Vertical do HIV, da Sífilis, da Hepatite B e da Doença de Chagas como problema de saúde pública^(2)^. Essas iniciativas, somadas à expansão da APS, especialmente por meio da Estratégia Saúde da Família, à sua articulação com a Vigilância em Saúde, à progressiva informatização dos serviços, à ampliação do uso do SINAN e à consolidação de rotinas de alimentação, crítica e monitoramento dos dados, podem ter favorecido a identificação mais oportuna dos casos, o acompanhamento longitudinal das crianças com SC, a sensibilização dos profissionais e a adoção de práticas mais sistemáticas de registro, contribuindo para a redução de campos ignorados ou em branco e para o aprimoramento gradual da qualidade da informação^(6,7)^. Embora não seja possível estabelecer relação causal entre essas iniciativas e os resultados observados, os achados sugerem que mudanças na organização dos serviços e nos processos de trabalho da vigilância e da atenção à saúde podem ter desempenhado um papel importante na melhoria da completude dos registros ao longo do período analisado.

Nesse sentido, a produção de informações de qualidade é condição essencial para subsidiar análises em saúde baseadas em evidências^(9)^. No caso da SC, doença evitável cuja eliminação está entre os compromissos internacionais do Brasil, são necessárias respostas coordenadas que integrem diagnóstico precoce, tratamento oportuno e seguimento rigoroso de recém-nascidos expostos ou acometidos^(3)^. Contudo, a efetividade dessas respostas depende da qualidade dos registros realizados pelos profissionais da rede de saúde, em especial pelos enfermeiros, que desempenham papel central na condução da vigilância na APS.

Apesar de progressos em variáveis clínicas e laboratoriais, como diagnóstico materno e testes no parto, a realização do TNT na mãe ainda requer atenção. Ainda que com baixa incompletude, essa variável é essencial para comparação com a sorologia não treponêmica da criança, orientando decisões diagnósticas, terapêuticas e de vigilância. Sua ausência compromete a condução clínica e a efetividade da resposta do sistema de saúde à SC^(3)^.

A variável tratamento da mãe demonstrou redução da incompletude entre os anos extremos, mas manteve percentuais elevados, sobretudo em comparação ao TNT. Esses achados evidenciam fragilidades persistentes em informações essenciais para o diagnóstico e a conduta clínica do recém-nascido e da criança^(15)^. Também persistiram níveis elevados de incompletude em campos, como ocupação e escolaridade materna, fundamentais para caracterizar o perfil sociodemográfico das gestantes. A ausência ou inadequação dessas informações compromete o monitoramento da SC e limita a implementação de estratégias direcionadas às populações mais vulneráveis^(2,16)^.

Embora o Ministério da Saúde, desde 2017, tenha deixado de considerar o tratamento da parceria sexual como critério de definição de caso de sífilis congênita para fins de notificação, essa mudança não eliminou a necessidade de seguimento de todas as crianças expostas ou com SC^(17)^. Nesse estudo, o tratamento das parcerias permaneceu como informação frequentemente incompleta ao longo da série histórica, sendo uma lacuna relevante, uma vez que o tratamento concomitante da mãe e das parcerias sexuais é essencial para interromper a transmissão do *Treponema pallidum* e prevenir reinfecções^(18)^. A baixa adesão das parcerias, associada ao desconhecimento da doença, à não realização do tratamento pelos serviços de saúde e às dificuldades institucionais, constitui importante entrave ao controle da transmissão vertical, pois mantém o risco de reinfecção e pode comprometer o tratamento oportuno e adequado da gestante. Assim, a ausência ou inadequação dessas informações reduz a capacidade analítica dos sistemas de informação para o acompanhamento da transmissão vertical^(3)^.

As variáveis relacionadas ao cuidado da criança apresentaram altos percentuais de campos em branco ou ignorados, especialmente em registros de LCR, testes laboratoriais pós-natais, radiografia de ossos longos e esquema terapêutico, evidenciando limitações importantes na completude dessas informações. Essas lacunas restringem a avaliação do diagnóstico conclusivo, do tratamento instituído, do seguimento e da individualização do cuidado, além de comprometerem a capacidade da vigilância de monitorar a condução clínica dos casos e possíveis manifestações tardias, muitas vezes silenciosas ou inespecíficas^(19)^.

Diante desse cenário, a incompletude dos registros de LCR e de testes laboratoriais assume relevância particular para a vigilância da neurossífilis congênita, forma grave e de difícil detecção, especialmente em recém-nascidos assintomáticos. Por poder manifestar-se em diferentes fases da infecção e com sinais variados, exige elevada suspeição clínica, mesmo na ausência de manifestações neurológicas evidentes^(20)^. Assim, a investigação criteriosa, o diagnóstico oportuno e o registro correto do esquema terapêutico são essenciais para assegurar o manejo e seguimento adequados da criança e identificar precocemente situações que demandem intervenção, contribuindo para prevenir alterações neurológicas, auditivas e do desenvolvimento psicomotor^(17)^. A literatura destaca que a completude das informações é fundamental para a adequada caracterização clínico-epidemiológica dos agravos e para subsidiar a avaliação das ações e dos serviços de saúde^(21)^.

Do ponto de vista da inconsistência, observaram-se incoerências entre variáveis interdependentes, como a relação entre diagnóstico clínico e presença de sinais e sintomas da criança, o que pode refletir falhas no registro das informações ou dificuldades na aplicação dos critérios de definição de caso.

Em relação ao seguimento da criança, os dados das FNI têm papel estratégico como instrumento da vigilância para o controle do agravo. Contudo, este estudo evidenciou elevada incompletude de algumas informações importantes: o TNT em sangue periférico, que deve ser realizado aos 1, 3, 6, 12 e 18 meses de vida, apresentou ausência de registro em 86,7% das FNI e, para o TT após 18 meses, o percentual de FNI sem essa informação foi de 99,3%. Conforme o PCDT do Ministério da Saúde^(3)^, os TNTs, como o VDRL (*Venereal Disease Research Laboratory*), são fundamentais na vigilância da criança após o nascimento, pois permitem confirmar o diagnóstico e monitorar a resposta ao tratamento pela quantificação dos títulos de anticorpos^(2,4)^. Assim, a ausência de dados sobre a titulação ascendente em TNTs subsequentes dificulta distinguir se houve falhas no registro do dado ou ausência de realização do teste. De modo semelhante, a ausência de informações sobre o TT após 18 meses pode refletir falhas no seguimento, limitações no registro, ou ambas, dificultando a confirmação diagnóstica da SC, conforme preconizado pelo protocolo ministerial^(3)^.

Além disso, foram identificadas duas limitações desse instrumento para o acompanhamento do seguimento da criança: a ausência de campos específicos para o registro do TNT em cada um dos momentos da série recomendada pelo Ministério da Saúde (1, 3, 6, 12 e 18 meses de vida) e o uso da opção “não se aplica” na variável referente à realização de TT após 18 meses, visto que esse exame é recomendado para confirmação ou descarte da infecção na criança e previsto no seguimento pelo protocolo vigente. Essas fragilidades podem comprometer a efetividade da FNI como instrumento de vigilância para seguimento da criança com SC.

Quanto às limitações do estudo, deve-se considerar a possibilidade de viés de informação, pois a incompletude dos campos pode refletir tanto falhas de registro quanto não realização do procedimento, sem possibilidade de diferenciá-las com base nas informações disponíveis no sistema. Destaca-se, ainda, que os dados foram incluídos até julho de 2021, último ano da série analisada. Entretanto, não foram identificadas políticas ou intervenções capazes de modificar significativamente os padrões de notificação nos meses subsequentes, de modo que os resultados permanecem representativos.

## CONCLUSÃO

O estudo avaliou a qualidade das FNI da SC como instrumento da vigilância epidemiológica para o seguimento da criança acometida pela sífilis congênita. Os resultados apontaram redução da incompletude dos campos da FNI de SC, mas persistência de inconsistências entre variáveis interdependentes e elevada incompletude dos campos relacionados aos resultados laboratoriais, comprometendo a avaliação da evolução clínica, do seguimento e da adequação da investigação das crianças com SC.

Essas limitações parecem decorrer não apenas de falhas no preenchimento dos registros, mas também de inadequações estruturais da ficha de notificação e investigação, cuja lógica transversal é insuficiente para representar um acompanhamento que se estende do nascimento aos 18 meses de vida. Embora a FNI contemple variáveis relacionadas ao monitoramento da SC, como a titulação ascendente do TNT e o resultado do TT após os 18 meses, ela não permite registrar sistematicamente todas as etapas do seguimento preconizadas pelos protocolos assistenciais. Além disso, o uso indistinto da categoria “não se aplica” nos campos de diagnóstico clínico e de teste treponêmico após os 18 meses dificulta diferenciar situações de não indicação clínica, não realização do exame e interrupção do acompanhamento, comprometendo a interpretação dos dados e a identificação de perdas de seguimento. Em conjunto, essas limitações reduzem o potencial analítico da vigilância epidemiológica e restringem o uso das informações para monitoramento dos casos, planejamento das ações e avaliação das estratégias de controle da sífilis congênita.

Diante disso, evidencia-se a necessidade de estratégias estruturantes para qualificar a notificação e a investigação da SC, incluindo capacitação permanente das equipes, supervisão sistemática dos registros e fortalecimento da integração entre vigilância em saúde, atenção primária e demais pontos da rede de atenção. Os resultados também indicam a necessidade de aperfeiçoamento da ficha do SINAN, com mecanismos que permitam registrar as diferentes etapas do monitoramento sorológico e a revisão das regras de preenchimento da categoria “não se aplica”.

Ressalta-se, por fim, que a qualidade da notificação e da investigação é essencial para que os sistemas de informação subsidiem a vigilância epidemiológica, o cuidado longitudinal e a tomada de decisão em saúde. Nesse sentido, a interoperabilidade entre sistemas e o uso de soluções digitais integradas, em consonância com os princípios do SUS Digital, constituem estratégias fundamentais para fortalecer a vigilância, qualificar o cuidado e ampliar a capacidade de resposta da rede de saúde à sífilis congênita.

## Data Availability

Os dados utilizados foram coletados no setor de Vigilância Epidemiológica da SESAB, no âmbito do Projeto Mãe, a partir do banco nominal do SINAN, referentes aos casos de SC no estado, entre 2010 e julho de 2021. As análises seguiram as metodologias estatísticas descritas neste manuscrito.

# Translation: Evaluación de la calidad de las notificaciones e investigaciones sobre la sífilis congénita en Bahía, 2010–2021

**DOI:** 10.1590/1980-220X-REEUSP-2025-0582es

**Authors:** Jeane Magnavita da Fonseca Cerqueira, Maria Aparecida Araújo Figueiredo, Nila Mara Smith Galvão, Monique Magnavita Borba da Fonseca Cerqueira, Magno Merces Weyll Pimentel

**Affiliations:** 1 Universidade do Estado da Bahia, Departamento de Ciência da Vida, Salvador, BA, Brazil.; 2 Universidade do Estado da Bahia, Departamento de Ciências Exatas e da Terra, Salvador, BA, Brazil.

**Keywords:** Sífilis Congénita, Servicios de Vigilancia Epidemiológica, Notificación de Enfermedades, Epidemiología, Sistemas de Información en Salud.

## Abstract

**Objetivo:**

Evaluar la calidad de la información contenida en las fichas de notificación e investigación de la sífilis congénita como herramienta de vigilancia epidemiológica para el seguimiento de los niños afectados por la enfermedad.

**Métodos:**

Estudio ecológico de tendencia temporal, con datos secundarios del SINAN de Bahía, entre 2010 y 2021. Se evaluaron los porcentajes de incompletitud anual, la variación porcentual proporcional y el porcentaje de inconsistencia. La tendencia temporal de la incompletitud se analizó mediante el coeficiente de correlación de Spearman.

**Resultados:**

De las diez variables maternas evaluadas, se observó una reducción de la falta de datos en siete de ellas. En los campos relativos al niño, se redujeron los grados de falta de datos en nueve de las once variables evaluadas (p ≤ 0,05). En el seguimiento del niño, el estudio puso de manifiesto que el 86,7 % de las fichas de investigación no contenían información sobre la prueba no treponémica en sangre periférica al nacer. En cuanto a la prueba treponémica realizada a los 18 meses, el porcentaje de fichas sin esta información fue del 99,3 %.

**Conclusión:**

A pesar de que se ha reducido el número de campos sin completar en la ficha de investigación de la sífilis congénita, los elevados porcentajes de fichas que carecen de información sobre los resultados de laboratorio dificultan la evaluación de la evolución y el seguimiento del niño con sífilis.

## INTRODUCCIÓN

La sífilis congénita (SC), de transmisión vertical, se considera el desenlace más grave de la infección debido a las posibles complicaciones para el niño. El diagnóstico es un reto, ya que muchos niños nacen asintomáticos o presentan signos clínicos leves e inespecíficos^(1)^. Esta característica afecta las estrategias de control, retrasando el inicio del tratamiento y dificultando el seguimiento adecuado^(2,3)^.

La evaluación del niño expuesto o afectado por la SC debe iniciarse inmediatamente después del nacimiento, con un estudio clínico, epidemiológico y de laboratorio, que se mantenga hasta los dos años de edad o, como mínimo, hasta los 18 meses^(2,3)^. El seguimiento longitudinal de todos los niños es fundamental para confirmar la curación definitiva e interrumpir el ciclo de transmisión. Sin embargo, la falta de exámenes de alta precisión para el diagnóstico en recién nacidos representa uno de los principales obstáculos para un monitoreo efectivo^(4)^. Además, se suma la necesidad de realizar una serie compleja de pruebas de laboratorio, especialmente los testes no treponémicos (TNT) en sangre periférica, cuya ejecución adecuada es esencial para el diagnóstico definitivo. La discontinuidad de estas pruebas compromete la comprensión epidemiológica y puede tener implicaciones para la salud del niño en etapas posteriores^(5)^.

En la SC, la atención continua exige la integración entre los diversos puntos de la red de atención de la salud infantil^(5)^. Una línea de atención estructurada permite un seguimiento calificado, en el que la vigilancia epidemiológica desempeña un papel estratégico en la identificación, notificación, investigación y monitoreo de los casos de SC^(6)^. Dado que la atención a los niños con SC comienza a partir del conocimiento del caso, en Brasil, la SC forma parte de la lista de enfermedades de notificación obligatoria en todo el territorio nacional desde 1986. El Sistema de Información sobre Enfermedades de Notificación Obligatoria (SINAN) es el principal instrumento para la recolección, el procesamiento y la distribución de información sobre enfermedades de notificación obligatoria, con base en los registros de los servicios de salud^(7)^. De esta manera, el seguimiento de los casos notificados hasta la conclusión de la investigación genera datos epidemiológicos que orientan las políticas y las acciones oportunas^(8)^.

En este sentido, la eficacia de la vigilancia de la SC depende de la calidad de la información, ya que los datos inconsistentes comprometen la confiabilidad de los indicadores y la implementación de medidas basadas en la evidencia^(9,10)^. El llenado adecuado de las notificaciones constituye una etapa esencial para la producción de información válida, y es resultado directo del trabajo de los profesionales de la salud en el día a día del servicio^(6)^.

En el ámbito de la salud pública, el enfermero se destaca en la integración entre la vigilancia y la Atención Primaria de Salud (APS), gracias a su formación generalista y su participación en la organización de los equipos, la gestión de la atención, el manejo clínico y el seguimiento epidemiológico de los casos de sífilis, lo que exige rigor técnico-científico, dominio de los protocolos y responsabilidad ética en el registro de la información. En este contexto, el llenado inadecuado de las Fichas de Notificación e Investigación (FNI) puede introducir sesgos en los sistemas de información, dificultar la comprensión del proceso de salud y enfermedad y la adopción de estrategias eficaces. Dado que se trata de un indicador de la calidad de la atención prenatal y materno-infantil, las fallas en estos registros pueden dar lugar a una subnotificación, comprometer la confiabilidad de los datos y repercutir directamente en la práctica del personal de enfermería de la APS^(10,11)^.

En el ámbito de la SC, el análisis continuo de los datos del SINAN es fundamental para obtener una visión fidedigna de la situación epidemiológica y para la planificación efectiva de las acciones de salud pública. Entre los principales criterios para evaluar la confiabilidad de los datos generados, se destacan la incompletitud, que se refiere a la falta de información necesaria, y la inconsistencia, que está relacionada con la coherencia entre campos interrelacionados^(10)^. Por lo tanto, las deficiencias en el monitoreo de la calidad de estos datos refuerzan la necesidad de investigaciones más exhaustivas^(12)^.

Desde esta perspectiva, el presente estudio tiene como objetivo evaluar la calidad de la información contenida en las fichas de notificación e investigación de la SC como instrumento de vigilancia epidemiológica en el seguimiento de los niños afectados por la enfermedad.

## MÉTODO

### Tipo de Estudio

Se trata de un estudio ecológico, de tendencia temporal, de los casos de SC notificados e investigados en el estado de Bahía, entre 2010 y 2021.

El estudio siguió las recomendaciones de la *Declaración RECORD* (*Reporting of studies Conducted using Observational Routinely-collected health Data*), una extensión de la declaración *STROBE* (*Strengthening the Reporting of Observational Studies in Epidemiology*), orientada a la estandarización y la transparencia en la realización y el informe de estudios con datos secundarios recopilados de manera rutinaria en sistemas de información en salud^(13,14)^.

### Lugar del Estudio

Estado de Bahía, Brasil, utilizando datos secundarios proporcionados por la Vigilancia Epidemiológica de la Secretaría de Salud del Estado de Bahía (SESAB).

### Población y Criterios de Selección

Se incluyeron todos los casos confirmados de SC registrados en el SINAN durante el período comprendido entre enero de 2010 y julio de 2021, de acuerdo con la definición nacional vigente. La SC es de notificación obligatoria, incluyendo casos de recién nacidos, mortinatos o abortos de mujeres con sífilis no tratada o tratada de manera inadecuada. El tratamiento se considera adecuado cuando se realiza con penicilina benzatina, según el estadio clínico, y se inicia hasta 30 días antes del parto^(2)^.

A efectos de vigilancia, se considera sífilis congénita (SC): I) los casos en niños menores de 13 años que presenten al menos una de las siguientes situaciones: manifestación clínica, del líquido cefalorraquídeo o radiológica de la enfermedad con TNT reactivo; títulos de TNT superiores a los de la madre en dos diluciones; títulos de TNT ascendentes en dos diluciones durante el seguimiento del niño expuesto; TNT reactivo después de los seis meses de edad, incluso con tratamiento neonatal adecuado; o Test Treponémico (TT) reactiva después de los 18 meses, sin diagnóstico previo de SC, excluyendo la sífilis adquirida; II) niño de hasta dos años con evidencia microbiológica de infección por *Treponema pallidum*, detectada en secreción nasal, lesión cutánea, biopsia o necropsia del niño, aborto o feto muerto al nacer, mediante exámenes directos con microscopía de campo oscuro o material teñido^(2)^.

### Definición de la Muestra o Cálculo del Tamaño de la Muestra

Al tratarse de un estudio censal basado en datos secundarios, no se realizó ningún cálculo muestral. Se analizaron todos los registros disponibles durante el período.

### Recolección de Datos

La FNI de SC cuenta con 66 campos, distribuidos entre gestantes/madres y niños. El segmento de la gestante/madre cuenta con 17 campos, distribuidos en antecedentes epidemiológicos, datos de laboratorio y tratamiento. La sección del niño incluye 20 campos, organizados en antecedentes epidemiológicos, datos de laboratorio y clínicos, tratamiento y evolución.

Las variables maternas analizadas fueron: edad, raza/color, ocupación, nivel educativo, asistencia a consultas prenatales, diagnóstico de sífilis, TNT y TT durante el parto o legrado, esquema de tratamiento y tratamiento de las parejas. En cuanto a los niños, se consideraron: municipio de residencia, TNT en sangre periférica, realización y fecha del TNT después de 18 meses, TNT en el líquido cefalorraquídeo (LCR), titulación ascendente del TNT, alteración del LCR, diagnóstico radiológico y clínico, signos y síntomas, esquema terapéutico, evolución y fecha del fallecimiento.

### Análisis y Procesamiento de los Datos

Los registros inconsistentes no se excluyeron, ya que constituían objeto de análisis del estudio. La identificación de duplicados se realizó con base en las variables “nombre de la madre” y “año de nacimiento del niño”, conservándose solo un registro por caso. Los registros referidos a embarazos gemelares se mantuvieron en la base de datos, ya que correspondían a eventos distintos.

Desde el punto de vista operativo, se consideró incompleto cuando el campo estaba en blanco o tenía el código “ignorado”; los campos con la categoría “no realizado” se consideraron completados, pero se analizaron por separado, ya que representaban una falla en la atención.

Para clasificar el grado de incompletitud, se adoptó la siguiente escala: ≤ 5 % de los campos incompletos (satisfactoriamente completo); de 5,1 a 10 % (poco incompleto); del 10,1 al 30 % (moderadamente incompleto); del 30,1 al 50 % (muy incompleto) y >50 % (altamente incompleto). Con base en esta clasificación, se evaluó la calidad de los datos a partir de los siguientes parámetros: i) porcentaje de incompletitud anual (proporción de campos no completados por año); ii) variación porcentual proporcional (VPP), calculada a partir de la variación proporcional del número absoluto de campos sin completar en el primer y último año de la serie; iii) porcentaje de inconsistencia (casos en los que se omitió o se completó incorrectamente una variable que depende de otra relacionada).

La tendencia temporal de la incompletitud se analizó mediante el coeficiente de correlación de Spearman (*r*_S_), un método no paramétrico adecuado para el análisis de la tendencia temporal de las proporciones de incompletitud, sin requerir una distribución normal de los datos. Considerando un nivel de significación estadística de *p* ≤ 0,05, la tendencia se clasificó como: i) creciente cuando el valor de *r*_S_ era positivo; ii) decreciente cuando el valor de *r*_S_ era negativo; y iii) estacionaria cuando el valor de *r*_S_ se encontraba cercano a cero.

Para el análisis de inconsistencias, se observó la relación entre los siguientes campos: i) diagnóstico clínico del niño marcado como asintomático; no aplicable; o ignorado *versus* el campo de presencia de signos y síntomas completado; ii) diagnóstico clínico del niño marcado como sintomático *versus* presencia de signos y síntomas, marcado como no aplicable o ignorado; iii) la realización de la prueba treponémica después de los 18 meses, marcada como reactiva o no reactiva *versus* la fecha de realización de la prueba antes de los 18 meses de edad (excepto abortos, mortinatos y fallecimientos). Los análisis se realizaron con el *software Stata*, versión 15.0.

### Aspectos Éticos

El estudio fue aprobado por el Comité de Ética en Investigación de la Universidad Estatal de Bahía (Dictamen n.º 7.619.184, CAAE 38510920. 6.0000.0057), con exención del Término de Consentimiento Libre e Informado por utilizar datos secundarios de dominio institucional, y fue financiado por la Fundación de Apoyo a la Investigación del Estado de Bahía (FAPESB), Convocatoria 02/2020.

### Uso de Inteligencia Artificial

Este manuscrito contó con el uso puntual de la herramienta de inteligencia artificial generativa ChatGPT (https://chat.openai.com), limitada al análisis de la coherencia y la fluidez textual. No se generó contenido científico ni referencias, y los análisis, interpretaciones y conclusiones fueron elaborados íntegramente por los autores, con verificación de las referencias en sus versiones completas.

## RESULTADOS

En el estado de Bahía, durante el período comprendido entre enero de 2010 y julio de 2021, se notificaron e investigaron 11 602 FNI de SC. El análisis de la tendencia de la incompletitud de estas FNI y el VPP entre el primer y el último año de la serie revelaron diferentes patrones y grados de incompletitud.

En los años observados, el análisis de tendencia del grado de incompletitud de diez variables maternas relacionadas con los niños con SC evidenció una reducción en siete de ellas, destacándose los siguientes campos: edad (VPP = –5,88; r_S_ *=* –0,76), raza/color (VPP = –42,42; r_S_ *=* –0,68), asistencia a la atención prenatal (VPP = –25,00; r_S_ *=* –0,80), diagnóstico durante la atención prenatal, el parto o el posparto (VPP = –13,63; r_S_ *=* –0,60), TT durante el parto (VPP = –36,11; r_S_ *=* –0,87), tratamiento de las parejas sexuales (VPP = –12,50; r_S_ *=* –0,68) y tratamiento de la madre (VPP = –28,57; r_S_ *=* –0,51). Excepto esta última, todas las demás tendencias mencionadas presentaron significación estadística (p ≤ 0,05) (Tabla 1).

**Tabla 1:** Tendencia de la incompletitud y variación porcentual proporcional entre el primer y el último año de la serie de las variables relacionadas con las madres, en las fichas de investigación de sífilis congénita – Bahía, Brasil, de enero de 2010 a julio de 2021.

Variables	2010	2011	2012	2013	2014	2015	2016	2017	2018	2019	2020	2021	VPP	*r*_s_	Valor de *p*
	Porcentaje de incompletitud														
Edad	5,36	11,54	10,19	7,54	5,68	4,00	3,84	3,63	2,62	3,68	1,84	4,41	–5,88	–0,76	0,004
Raza/color	20,82	28,74	24,60	18,86	10,31	12,69	12,85	15,75	17,64	17,96	9,58	10,47	–42,42	–0,68	0,015
Ocupación	41,96	38,87	31,63	34,31	42,39	43,19	41,97	45,72	37,76	39,93	36,87	40,77	11,27	0,07	0,820
Nivel educativo	41,96	41,50	40,42	45,50	43,55	42,33	36,73	30,77	33,87	39,01	41,34	42,15	15,03	–-0,32	0,307
Ejecución del PN	20,19	20,04	21,09	21,41	16,09	19,85	11,45	10,89	8,50	8,77	10,22	13,22	–25,00	–0,80	0,000
Diagnóstico de sífilis en el embarazo, el parto y el puerperio	13,88	13,56	16,17	11,44	8,00	8,01	8,24	7,26	5,88	6,43	9,58	10,47	–13,63	–0,60	0,038
TNT cualitativo en el parto	5,68	7,49	9,14	6,93	4,91	4,68	6,08	5,30	4,79	3,76	2,63	4,96	0,00	–0,70	0,011
TT durante el parto	22,71	19,84	24,25	22,14	15,51	19,51	19,13	15,53	12,33	11,53	15,24	12,67	–36,11	–0,87	0,000
Tratamiento de la madre	26,50	17,41	24,78	20,19	15,51	14,99	15,64	14,95	19,81	15,96	15,16	16,53	–28,57	–0,51	0,080
Tratamiento de las relaciones sexuales	37,85	32,19	41,65	45,26	28,42	29,22	27,44	13,06	12,97	27,32	27,45	28,93	–12,50	–0,68	0,010

Nota: PN – Prenatal; TNT – Prueba no treponémica; TT – Prueba treponémica; VPP – Variación porcentual proporcional; r_s_ – Coeficiente de correlación de Spearman.

En cuanto a los grados de incompletitud analizados por año, la variable “edad” se mantuvo poco incompleta (del 5,1 % al 10 %), excepto en 2011; la variable “raza/color” fue moderadamente incompleta (del 10,1 % al 30 %), reduciéndose del 20,82 % al 15,59 %; y la ocupación y el nivel educativo de la madre se mantuvieron muy incompletos (del 30,1 % al 50 %) durante toda la serie. La asistencia a la atención prenatal fue moderadamente incompleta (del 10,1 % al 30 %), pasando a ser poco incompleta en 2018 y 2019 (del 5,1 % al 10 %); sin embargo, el diagnóstico de sífilis materna mejoró a partir de 2014, clasificándose como poco incompleto (del 5,1 % al 10 %). Los campos relacionados con el TNT durante el parto fueron poco incompletos (del 5,1 % al 10 %) en la mayoría de los años, alcanzando un grado satisfactoriamente completo (≤ 5 %) en 2014 y 2015 y de 2018 a 2021, pero los campos del TT se mantuvieron moderadamente incompletos (del 10,1 % al 30 %). El tratamiento de la madre varió de moderadamente incompleto (del 10,1 % al 30 %) a poco incompleto (del 5,1 % al 10 %), mientras que el tratamiento de la pareja pasó de ser muy incompleto (del 30,1 % al 50 %) a moderadamente incompleto (del 10,1 % al 30 %), a partir de 2014 (Tabla 1). Las variables de antecedentes epidemiológicos del niño presentaron un grado satisfactoriamente completo (< 5 %) en los campos “semana epidemiológica”, “fecha de llenado de la notificación”, “edad” y “sexo” (datos no mostrados).

El análisis de la tendencia de incompletitud de 11 variables relacionadas con los antecedentes epidemiológicos del niño con SC mostró que, entre 2010 y 2021, se produjo una reducción constante en los grados de incompletitud en nueve de ellas, todas con significación estadística (p ≤ 0,05), a saber: alteración del análisis del LCR (VPP = –26,03; r_S_ = –0,60), TNT en el LCR (VPP = –41,35; r_S_ = –0,91), TNT en sangre periférica (VPP = –48,80; r_S_ = –0,71), diagnóstico clínico (VPP = –53,03; r_S_ = –0,94), diagnóstico radiológico (VPP = –27,50; r_S_ = –0,93), esquema de tratamiento (VPP = –21,21; r_S_ = –0,92), TT tras 18 meses (VPP = –30,46; r_S_ = –0,84), fecha de la TT tras 18 meses (VPP = –28,77; r_s_ = –0,88), fecha del fallecimiento (VPP = –37,04; r_S_ = –0,60) (Tabla 2).

**Tabla 2:** Tendencia de la incompletitud y variación porcentual proporcional entre el primer y el último año de la serie en la cumplimentación de las variables relacionadas con el niño, en las fichas de investigación de la sífilis congénita – Bahía, Brasil, de enero de 2010 a julio de 2021.

Variables	2010	2011	2012	2013	2014	2015	2016	2017	2018	2019	2020	2021	VPP	*r*_s_	Valor de *p*
	Porcentaje de datos incompletos														
Lugar de nacimiento	5,36	19,23	24,25	17,40	15,13	12,86	9,71	12,26	10,42	4,18	3,99	5,23	0,00	–0,70	0,011
Alteraciones en el análisis del LCR	46,06	49,39	53,95	47,81	43,93	36,97	31,22	33,02	31,05	27,07	21,39	29,75	–26,03	–0,60	0,000
TNT en LCR	41,96	45,95	49,74	44,04	42,00	35,35	27,58	28,74	25,69	25,06	17,16	21,49	–41,35	–0,91	0,000
TNT en sangre	12,93	28,95	30,40	22,99	18,40	17,89	14,53	15,67	13,74	8,10	8,06	11,85	–48,80	–0,71	0,009
Diagnóstico clínico	41,64	49,19	40,42	30,29	28,71	21,21	21,72	22,06	19,23	14,12	13,97	17,08	–53,03	–0,94	0,000
Diagnóstico radiológico	50,47	52,92	58,52	49,88	49,52	40,20	32,40	34,62	34,12	27,74	25,70	31,96	–27,50	–0,93	0,000
Esquema de tratamiento	91,70	29,96	32,51	25,19	21,39	19,08	18,23	18,51	17,89	11,19	11,89	14,60	–21,21	–0,92	0,000
TT tras 18 meses	40,38	45,14	48,33	42,94	36,03	32,96	24,09	25,54	28,95	29,32	18,91	24,52	–30,46	–0,84	0,000
Fecha de la TT tras 18 meses	43,85	46,36	49,74	46,11	43,74	39,86	28,07	27,50	30,86	31,24	22,19	27,27	–28,77	–0,88	0,000
Evolución	15,14	14,98	17,05	17,60	15,30	9,20	10,27	15,46	14,38	9,02	11,09	9,09	–31,25	–0,50	0,095
Fecha del fallecimiento	17,03	17,00	19,68	19,46	16,74	10,82	11,38	16,55	15,08	9,36	11,97	9,37	–37,04	–0,80	0,039

Nota: TNT – Prueba no treponémica; TT – Prueba treponémica; VPP – Variación porcentual proporcional; r_s_ – Coeficiente de correlación de Spearman.

El análisis del grado de incompletitud por año puso de manifiesto que el campo «municipio de residencia» osciló entre «poco incompleto» (del 5,1 % al 10 %) en los años 2010, 2019, 2020 y 2021, y «moderadamente incompleto» (del 10,1 % al 30 %) en el resto de años. El TNT en el líquido cefalorraquídeo presentó un grado muy incompleto (del 30,1 % al 50 %) hasta 2015, reduciéndose posteriormente a moderadamente incompleto (del 10,1 % al 30 %). Por su parte, el TNT en sangre periférica se consideró muy incompleto (del 30,1 % al 50 %) en la mayoría de los años, excepto en 2019 y 2020, cuando pasó a ser poco incompleto (del 5,1 % al 10 %). El campo «alteraciones en el LCR» se mantuvo muy incompleto (del 30,1 % al 50 %) en la mayoría de los años, llegando a ser altamente incompleto (> 50 %) en 2012; sin embargo, en los tres últimos años de la serie, pasó a ser moderadamente incompleto (del 10,1 % al 30 %). El diagnóstico radiológico fue muy incompleto en los tres primeros años (> 50 %), pasando a ser muy incompleto (del 30,1 % al 50 %) en los seis años siguientes y moderadamente incompleto (del 5,1 % al 10 %) en 2019 y 2020. El esquema de tratamiento se mantuvo moderadamente incompleto (del 10,1 % al 30 %) durante la mayor parte del periodo. El TT tras 18 meses varió de muy incompleto (del 30,1 % al 50 %) entre 2010 y 2015 a moderadamente incompleto (del 10,1 % al 30 %) en los años siguientes. La fecha de realización del TT tras 18 meses osciló entre muy incompleta (del 30,1 % al 50 %) y moderadamente incompleta (del 10,1 % al 30 %). El campo «evolución del caso» alternó entre moderadamente incompleto (del 10,1 % al 30 %) y poco incompleto (del 5,1 % al 10 %), mientras que el campo «fecha del fallecimiento» fue moderadamente incompleto (del 10,1 % al 30 %) durante la mayor parte del período y ligeramente incompleto (del 5,1 % al 10 %) en tres de los 12 años estudiados. El campo «signos y síntomas» de los niños con SC osciló entre moderadamente incompleto (del 10,1 % al 30 %), muy incompleto (del 30,1 % al 50 %) y altamente incompleto (> 50 %). El campo de la variable «asintomático» osciló entre muy incompleto (del 30,1 % al 50 %) y moderadamente incompleto (del 10,1 % al 30 %) (Tabla 2).

La Tabla 3 presenta el número de signos y síntomas registrados en las fichas de investigación de la sífilis congénita, según el diagnóstico clínico. De los 11 602 registros analizados, el 61,45 % (7 130/11,602) se diagnosticaron como asintomáticos, el 10 % (1 161/11 602) como sintomáticos, el 5,06 % (588/11 602) como «no aplicable» y el 13,05 % (1 515/11 602) como desconocidos o en blanco. Entre los clasificados como sintomáticos (1.161), el 21,27 % (247/1.161) no presentaba signos ni síntomas registrados. Entre los clasificados como asintomáticos, seis presentaban signos y síntomas registrados.

**Tabla 3:** Número de signos y síntomas registrados en las fichas de investigación de la sífilis congénita, por diagnóstico clínico – Bahía, Brasil, de enero de 2010 a julio de 2021.

Diagnóstico clínico	0	1	2	3	4	5	6	7	8	Total
Asintomáticos	7 124	6	0	0	0	0	0	0	0	7 130
Sintomático	247	707	110	53	24	10	3	3	4	1 161
No aplicable	588	0	0	0	0	0	0	0	0	588
Ignorado	1 515	0	0	0	0	0	0	0	0	1 515
En blanco	1 208	0	0	0	0	0	0	0	0	1 208
Total	10 682	713	110	53	24	10	3	3	4	11 602

Fuente: SINAN.

La primera parte de la Tabla 4 compara el TNT en sangre periférica al nacer con los datos introducidos en el campo «titulación ascendente» en las pruebas posteriores, que deben realizarse en los meses 1, 3, 6, 12 y 18. De las 8 360 FNI de SC en niños vivos, 4 765 no se sometieron a la prueba para observar la titulación posterior y 2 484 presentaban una situación desconocida, lo que supone un total del 86,7 % de las FNI de SC sin esta información. En la segunda parte de la Tabla 4 se muestra la situación del campo «TT realizada después de 18 meses», teniendo en cuenta la edad del niño en la fecha de la prueba. De los 8.178 casos de FNI de SC en niños vivos, 8.121 (99,3 %) carecían de información sobre la realización de la prueba, dos se sometieron a la prueba antes de los 18 meses y 56 (0,7 %) se sometieron a la prueba a la edad recomendada (más de 18 meses).

**Tabla 4:** Cumplimentación del campo «titulación ascendente» relacionado con la prueba no treponémica y situación de la prueba treponémica tras los 18 meses, según la edad del niño en la fecha de realización de la prueba, en las fichas de investigación de sífilis congénita – Bahía, Brasil, de enero de 2010 a julio de 2021.

Prueba no treponémica en sangre periférica	Titulación ascendente					
	Sí	No	No se ha realizado	Se desconoce	En blanco	Total
Reactivo	94	1 017	4 765	2.484	0	8 360
No reactivo	4	68	455	166	0	693
No realizado	7	11	619	85	0	722
Ignorado	6	18	154	443	0	621
En blanco	0	0	0	0	1.206	1 206
Total	111	1.114	5 993	3.178	1.206	11 602
**Prueba treponémica tras 18 meses**	**Edad de los niños vivos en el momento de la prueba**					
	**<18 meses**		**De 18 a 24 meses**	**>24 meses**	**Sin datos**	**Total**
Reactivo	0		1	4	162	167
No reactivo	2		26	24	139	191
No realizado	0		0	0	2 418	2 418
No aplicable	0		0	0	3 097	3.097
Sin datos	0		0	0	1 576	1 576
En blanco	0		0	0	729	729
Total	2		27	28	8 121	8.178

Fuente: SINAN.

## DISCUSIÓN

En el estado de Bahía, la tendencia a la incompletitud de los FNI de SC, desde enero de 2010 hasta julio de 2021, se redujo en la mayoría de las variables analizadas. Sin embargo, persistieron inconsistencias en variables esenciales para el seguimiento de los niños con infección, especialmente aquellas relacionadas con los análisis de laboratorio indispensables para el seguimiento clínico-laboratorial y la evaluación de la respuesta terapéutica. Estos hallazgos señalan debilidades en la calidad de los registros y limitan el seguimiento de la evolución clínica y laboratorial de los casos.

La mejora en la exhaustividad de algunas de las variables a lo largo de la serie histórica podría estar relacionada con cambios en la organización de la vigilancia de la sífilis congénita en Brasil, con el fortalecimiento de las políticas públicas orientadas a la eliminación de la transmisión vertical y con la mejora de la calidad de la vigilancia y la atención, incluyendo la implementación del Protocolo Clínico y las Directrices Terapéuticas (PCDT) para la Atención Integral a las Personas con Infecciones de Transmisión Sexual y del Pacto Nacional para la Eliminación de la Transmisión Vertical del VIH, la Sífilis, la Hepatitis B y la Enfermedad de Chagas como problema de salud pública^(2)^. Estas iniciativas, sumadas a la expansión de la atención primaria de salud (APS), especialmente a través de la Estrategia de Salud Familiar, a su articulación con la vigilancia en salud, a la informatización progresiva de los servicios, a la ampliación del uso del SINAN y a la consolidación de rutinas de alimentación, análisis crítico y monitoreo de los datos, pueden haber favorecido la identificación más oportuna de los casos, el seguimiento longitudinal de los niños con SC, la sensibilización de los profesionales y la adopción de prácticas de registro más sistemáticas, lo que ha contribuido a reducir los campos ignorados o en blanco y a mejorar gradualmente la calidad de la información^(6,7)^. Aunque no es posible establecer una relación causal entre estas iniciativas y los resultados observados, los hallazgos sugieren que los cambios en la organización de los servicios y en los procesos de trabajo de la vigilancia y la atención de la salud pueden haber desempeñado un papel importante en la mejora de la exhaustividad de los registros a lo largo del período analizado.

En este sentido, la generación de información de calidad es una condición esencial para respaldar los análisis en salud basados en la evidencia^(9)^. En el caso de la SC, una enfermedad prevenible cuya eliminación figura entre los compromisos internacionales de Brasil, se requieren respuestas coordinadas que integren el diagnóstico precoz, el tratamiento oportuno y el seguimiento riguroso de los recién nacidos expuestos o afectados^(3)^. Sin embargo, la efectividad de estas respuestas depende de la calidad de los registros realizados por los profesionales de la red de salud, en especial por los enfermeros, quienes desempeñan un papel central en la vigilancia en la atención primaria de salud.

A pesar de los avances en variables clínicas y de laboratorio, como el diagnóstico materno y las pruebas durante el parto, la realización de la prueba de TNT en la madre aún requiere atención. Aunque presenta un bajo nivel de incompletitud, esta variable es esencial para la comparación con la serología no treponémica del niño, ya que orienta las decisiones diagnósticas, terapéuticas y de vigilancia.

Su ausencia compromete el manejo clínico y la eficacia de la respuesta del sistema de salud ante la SC^(3)^.

La variable “tratamiento de la madre” mostró una reducción de la falta de datos entre los años extremos, pero mantuvo porcentajes elevados, sobre todo en comparación con la prueba TNT. Estos hallazgos evidencian debilidades persistentes en la información esencial para el diagnóstico y el manejo clínico del recién nacido y del niño^(15)^. También persistieron altos niveles de información incompleta en campos como la ocupación y el nivel educativo de la madre, fundamentales para caracterizar el perfil sociodemográfico de las gestantes. La ausencia o la insuficiencia de esta información compromete el seguimiento de la SC y limita la implementación de estrategias dirigidas a las poblaciones más vulnerables^(2,16)^.

Aunque el Ministerio de Salud, desde 2017, dejó de considerar el tratamiento de la pareja sexual como criterio para definir un caso de sífilis congénita a efectos de notificación, este cambio no eliminó la necesidad de dar seguimiento a todos los niños expuestos o con SC^(17)^. En este estudio, la información sobre el tratamiento de las parejas siguió siendo a menudo incompleta a lo largo de la serie histórica, lo que constituye una laguna importante, ya que el tratamiento simultáneo de la madre y de las parejas sexuales es esencial para interrumpir la transmisión de *Treponema pallidum* y prevenir las reinfecciones^(18)^. La baja adherencia de las parejas, asociada al desconocimiento de la enfermedad, a la falta de tratamiento por parte de los servicios de salud y a las dificultades institucionales, constituye un obstáculo importante para el control de la transmisión vertical, ya que mantiene el riesgo de reinfección y puede comprometer el tratamiento oportuno y adecuado de la mujer embarazada. Así, la ausencia o la insuficiencia de esta información reduce la capacidad analítica de los sistemas de información para el seguimiento de la transmisión vertical^(3)^.

Las variables relacionadas con el cuidado del niño presentaron altos porcentajes de campos en blanco o ignorados, especialmente en los registros de LCR, pruebas de laboratorio posnatales, radiografía de huesos largos y esquema terapéutico, lo que pone de manifiesto importantes limitaciones en la exhaustividad de esta información. Estas lagunas limitan la evaluación del diagnóstico definitivo, del tratamiento instituido, del seguimiento y de la atención individualizada, además de comprometer la capacidad de la vigilancia para monitorear el manejo clínico de los casos y las posibles manifestaciones tardías, que a menudo son asintomáticas o inespecíficas^(19)^.

Ante este panorama, la incompletitud de los registros de LCR y de los análisis de laboratorio cobra especial relevancia para la vigilancia de la neurosífilis congénita, una forma grave y de difícil detección, especialmente en recién nacidos asintomáticos. Dado que puede manifestarse en diferentes fases de la infección y con signos variados, exige un alto grado de sospecha clínica, incluso en ausencia de manifestaciones neurológicas evidentes^(20)^. Por lo tanto, la investigación minuciosa, el diagnóstico oportuno y el registro correcto del esquema terapéutico son esenciales para garantizar el manejo y seguimiento adecuados del niño e identificar de manera temprana situaciones que requieran intervención, lo que contribuye a prevenir alteraciones neurológicas, auditivas y del desarrollo psicomotor^(17)^.

La literatura destaca que la exhaustividad de la información es fundamental para la caracterización clínico-epidemiológica adecuada de las afecciones y para respaldar la evaluación de las acciones y los servicios de salud^(21)^.

Desde el punto de vista de la inconsistencia, se observaron incoherencias entre variables interdependientes, como la relación entre el diagnóstico clínico y la presencia de signos y síntomas en el niño, lo que puede reflejar fallas en el registro de la información o dificultades en la aplicación de los criterios de definición de caso.

En cuanto al seguimiento del niño, los datos de las FNI desempeñan un papel estratégico como instrumento de vigilancia para el control de la afección. Sin embargo, este estudio puso de manifiesto una elevada falta de información en algunos datos importantes: la prueba TNT en sangre periférica, que debe realizarse a los 1, 3, 6, 12 y 18 meses de vida, no se registró en el 86,7 % de las FNI y, en cuanto a la prueba TT después de los 18 meses, el porcentaje de FNI sin esta información fue del 99,3 %. Según el PCDT del Ministerio de Salud^(3)^, las pruebas de titulación de anticuerpos (TNT), como el VDRL (*Venereal Disease Research Laboratory*), son fundamentales para el seguimiento del niño después del nacimiento, ya que permiten confirmar el diagnóstico y monitorear la respuesta al tratamiento mediante la cuantificación de los títulos de anticuerpos^(2,4)^. Por lo tanto, la falta de datos sobre la titulación ascendente en las pruebas TNT posteriores dificulta distinguir si hubo fallas en el registro de los datos o si no se realizó la prueba. De manera similar, la falta de información sobre la prueba TT a los 18 meses puede reflejar fallas en el seguimiento, limitaciones en el registro o ambas cosas, lo que dificulta la confirmación diagnóstica de la SC, tal como lo recomienda el protocolo ministerial^(3)^.

Además, se identificaron dos limitaciones de este instrumento para el seguimiento del niño: la ausencia de campos específicos para registrar la prueba de TNT en cada uno de los momentos de la serie recomendada por el Ministerio de Salud (1, 3, 6, 12 y 18 meses de vida) y el uso de la opción “no aplica” en la variable referente a la realización de la prueba de TT después de los 18 meses, dado que este examen se recomienda para confirmar o descartar la infección en el niño y está previsto en el seguimiento según el protocolo vigente. Estas deficiencias pueden comprometer la efectividad de la FNI como instrumento de vigilancia para el seguimiento del niño con SC.

En cuanto a las limitaciones del estudio, se debe considerar la posibilidad de un sesgo de información, ya que los campos incompletos pueden reflejar tanto fallas en el registro como la no realización del procedimiento, sin posibilidad de diferenciarlas con base en la información disponible en el sistema. Cabe destacar, además, que los datos se incluyeron hasta julio de 2021, último año de la serie analizada. Sin embargo, no se identificaron políticas ni intervenciones capaces de modificar significativamente los patrones de notificación en los meses posteriores, por lo que los resultados siguen siendo representativos.

## CONCLUSIÓN

El estudio evaluó la calidad de los FNI de la sífilis congénita como instrumento de vigilancia epidemiológica para el seguimiento de los niños afectados por esta enfermedad. Los resultados señalaron una reducción de la falta de información en los campos de la FNI de SC, pero también la persistencia de inconsistencias entre variables interdependientes y una elevada falta de información en los campos relacionados con los resultados de laboratorio, lo que compromete la evaluación de la evolución clínica, el seguimiento y la adecuación de la investigación de los niños con SC.

Estas limitaciones parecen deberse no solo a fallas en el llenado de los registros, sino también a deficiencias estructurales de la ficha de notificación e investigación, cuya lógica transversal es insuficiente para representar un seguimiento que se extiende desde el nacimiento hasta los 18 meses de vida. Aunque la FNI incluye variables relacionadas con el monitoreo de la SC, como la titulación ascendente del TNT y el resultado del TT después de los 18 meses, no permite registrar sistemáticamente todas las etapas del seguimiento recomendadas por los protocolos de atención. Además, el uso indiscriminado de la categoría “no aplica” en los campos de diagnóstico clínico y de prueba treponémica después de los 18 meses dificulta diferenciar entre situaciones de no indicación clínica, no realización de la prueba e interrupción del seguimiento, lo que compromete la interpretación de los datos y la identificación de pérdidas de seguimiento. En conjunto, estas limitaciones reducen el potencial analítico de la vigilancia epidemiológica y restringen el uso de la información para el monitoreo de los casos, la planificación de acciones y la evaluación de las estrategias de control de la sífilis congénita.

Ante esto, se pone de manifiesto la necesidad de estrategias estructurales para mejorar la notificación y la investigación de la sífilis congénita, incluyendo la capacitación continua de los equipos, la supervisión sistemática de los registros y el fortalecimiento de la integración entre la vigilancia en salud, la atención primaria y otros puntos de la red de atención. Los resultados también indican la necesidad de perfeccionar el formulario del SINAN, con mecanismos que permitan registrar las diferentes etapas del seguimiento serológico y la revisión de las reglas para completar la categoría “no aplica”.

Por último, cabe destacar que la calidad de la notificación y la investigación es esencial para que los sistemas de información respalden la vigilancia epidemiológica, la atención longitudinal y la toma de decisiones en materia de salud. En este sentido, la interoperabilidad entre sistemas y el uso de soluciones digitales integradas, en consonancia con los principios del SUS Digital, constituyen estrategias fundamentales para fortalecer la vigilancia, mejorar la atención y ampliar la capacidad de respuesta de la red de salud ante la sífilis congénita.

## Data Availability

Los datos utilizados se recopilaron en el sector de Vigilancia Epidemiológica de la SESAB, en el marco del Proyecto Madre, a partir de la base de datos nominal del SINAN, referidos a los casos de sífilis congénita en el estado, entre 2010 y julio de 2021. Los análisis siguieron las metodologías estadísticas descritas en este manuscrito.
